# Modular delivery of co-stimulatory signals through a PD-1-based immunoswitch receptor improves the functionality of Hepatitis B Virus-specific engineered T cells

**DOI:** 10.3389/fimmu.2026.1821414

**Published:** 2026-07-03

**Authors:** Luis Felipe Olguín-Contreras, Johanna Heep, Lisa Schiller, Eva Loffredo-Verde, Marvin M. Festag, Kai Metzger, Stephanie Färber, Merve Gültan, Margaret Tulessin, Susanne Wilde, Karin Wisskirchen, Elfriede Noessner, Ulrike Protzer

**Affiliations:** 1 Institute of Virology, Technical University of Munich, School of Medicine and Health, Munich, Germany; 2 Institute of Pathology, Technical University of Munich, School of Medicine and Health, Munich, Germany; 3 SCG Cell Therapy GmbH, Munich, Germany; 4 Immunanalytics-Tissue Control of Immunocytes, Helmholtz Center Munich, Munich, Germany; 5 Institute of Virology, Helmholtz Center Munich, Munich, Germany

**Keywords:** CAR-T cells, chronic hepatitis B, co-stimulatory domains, hepatocellular carcinoma, immune checkpoints, PD-1/PD-L1 signaling, T-cell exhaustion, TCR-T cells

## Abstract

In chronic hepatitis B virus infection (cHBV), T-cell responses are skewed. This prevents the elimination of HBV-infected hepatocytes, contributes to hepatocellular carcinoma development, and limits the efficacy of T-cell therapies. To counteract the immune checkpoint PD-1/PD-L1–interaction in adoptive T-cell therapy, we developed PD-1–based immunoswitch receptors that convert PD-L1 engagement into co-stimulation. We designed immunoswitch receptors linking a PD-1 ectodomain to intracellular CD28, 4-1BB, or OX40 signaling domains and expressed them in HBV-specific chimeric antigen receptor (CAR)- and T-cell receptor-engineered T cells. T-cell activity was assessed in antigen- and PD-L1-dependent co-cultures, transcription factor reporter systems, and an HBV carrier mouse model. Second-generation S-CAR functionality was not improved by signal stacking of additional co-stimulatory domains, resulting in a dysfunctional activation state rather than an additive benefit. In contrast, modular delivery of co-stimulation through PD-1-based immunoswitch receptors enhanced antigen sensitivity, functionality, and cytotoxicity of engineered T cells. PD-L1 supplied in trans was sufficient to support on-target functionality of engineered T cells by immunoswitch receptor co-stimulation. PD-1_4-1BB proved superior at inducing sustained NF-κB signaling and reducing exhaustion-associated pathways. *In vivo*, PD-1_4-1BB improved T-cell persistence and decreased TOX upregulation. PD-1–based immunoswitch receptors offer a modular strategy to strengthen engineered T-cell responses against HBV antigen-expressing cells in the liver’s tolerizing environment. This ligand-driven approach enables precise tuning of co-stimulation, generating a versatile platform for enhancing adoptive T-cell therapies in chronic infections and cancer.

## 1 Introduction

Chronic hepatitis B virus infection (cHBV) remains one of the world’s most persistent viral threats, affecting more than 250 million people globally and contributing to approximately half of all hepatocellular carcinoma (HCC) cases. Despite significant advances in antiviral therapy and preventive vaccination, chronic HBV infection continues to impose a substantial clinical and socioeconomic burden, causing 1.1 million deaths per year (1–3). A defining feature of this infection is the virus’s remarkable ability to evade immune clearance. Central to HBV persistence is its genomic reservoir, covalently closed circular DNA (cccDNA), which remains stable within hepatocytes and resists current therapeutic approaches (4, 5). As a result, most patients require lifelong antiviral treatment yet rarely achieve a functional cure. In addition, HBV can integrate into the host cell genome over time. HBV integration prevents the formation of new progeny viruses, but hepatocytes carrying the integrated HBV-DNA still express HBV antigens that can serve as T-cell targets.

HBV is a non-cytolytic virus that can persist in an infected hepatocyte for years. Chronic HBV infection profoundly alters the host’s immune response against antigen-positive cells. Persistent high-level viral antigen exposure, coupled with the liver’s inherently tolerogenic environment, drives a progressive decline in HBV-specific CD8+ and CD4+ T-cell numbers and function (6, 7). This deterioration manifests as reduced proliferative capacity, diminished cytokine production, impaired cytotoxicity, the stepwise development of T-cell exhaustion, and a depletion of T-cell clones (7). Inhibitory receptors such as PD-1, TIM-3, LAG-3, and TIGIT are upregulated on HBV-specific T cells in chronically infected individuals, often correlating with viral load and disease severity (8, 9). Among these, the PD-1/PD-L1 axis plays a dominant role in suppressing T-cell activity in both the chronically infected liver and HBV-driven HCC (8, 10). PD-L1 is widely expressed not only by infected hepatocytes but also by liver sinusoidal endothelial cells, Kupffer cells, hepatic stellate cells, and HCC tumor cells—creating a microenvironment that reinforces immune exhaustion and restrains antiviral surveillance (10–12). Thus, HBV persistence is a consequence of a finely tuned ecosystem that prevents the elimination of HBV-positive cells with remarkable efficiency.

Adoptive T-cell therapies, including chimeric antigen receptor (CAR)-T cells and T-cell receptor (TCR)-engineered T cells, have emerged as powerful approaches to restore antiviral immunity in chronic infections. Several HBV-specific CAR- and TCR-constructs have demonstrated potent cytolytic activity *in vitro* (13, 14) and in preclinical models (15–21). Their translational appeal lies in their ability to provide fresh T cells to the dysfunctional endogenous T-cell repertoires and directly target infected hepatocytes or cancer cells that present HBV antigens (22). However, despite this promise, therapeutic efficacy remains limited by the same microenvironmental constraints that impair natural antiviral immunity. Engineered T cells entering the chronically infected liver encounter high antigen burden, low co-stimulatory support, and dominant inhibitory signaling (22). As a result, they often experience rapid dysfunction, early exhaustion, and attrition, mirroring the fate of endogenous HBV-specific T cells (23, 24).

Second- and third-generation CAR designs incorporate co-stimulatory domains such as CD28, 4-1BB, or OX40 to strengthen activation, enhance survival, and increase cytokine production (25–27). Yet simply adding extra co-stimulatory domains to the CAR structure does not necessarily yield better outcomes. In HBV-specific CAR T cells, stacking CD28 with 4-1BB or OX40 did not improve functionality and, in some cases, even impaired performance (28). These observations suggest that engineered T-cell failure in chronic HBV infection is not merely a matter of insufficient activation, but of improper spatial and contextual integration of signals. T-cell activation is naturally orchestrated through the coordinated engagement of the TCR, co-stimulatory molecules, and cytokine receptors at the immunological synapse. This spatially controlled system is difficult to replicate by embedding multiple signaling domains into a single receptor. Moreover, in the liver, where antigen and inhibitory ligands are spatially segregated among hepatocytes, endothelial cells, and myeloid populations, engineered T cells may lack access to appropriately positioned co-stimulatory signals (12). Thus, next-generation strategies must move beyond “more signal” toward “correctly delivered signal.”.

To address these limitations, we developed PD-1-based immunoswitch receptors, which serve as a modular receptor platform designed to exploit PD-L1 (typically an inhibitory ligand) as a source of controlled positive co-stimulation (29–32). These immunoswitches consist of the PD-1 extracellular domain fused to different intracellular co-stimulatory modules, including CD28, 4-1BB, or OX40. This design serves three strategic purposes: 1) Preventing engagement of endogenous PD-1, thereby blocking inhibitory signaling. 2) Converting PD-L1 expression in the chronically infected and tumorous liver into a driver of T-cell activation. 3) Separating the processes of co-stimulation from antigen recognition and placing it under the control of an independently expressed ligand. Thus, immunoswitch receptors re-establish a more physiological topology of T-cell activation. Rather than overloading CAR- or TCR-constructs, immunoswitches distribute activation across multiple receptors, enabling a more balanced tuning of T-cell performance.

In this study, we systematically examine the functional impact of PD-1–based immunoswitch receptors in HBV-specific CAR- and TCR-T cells. Using co-cultures of engineered primary T cells with antigen-expressing target cells, transcriptional reporter assays, and an *in vivo* chronic HBV-carrier mouse model, we characterize how these modular co-stimulatory inputs reshape T-cell activation, cytokine production, cytotoxicity, and exhaustion trajectories. This allowed us to distinguish mechanistic differences among the CD28, 4-1BB, and OX40 signaling variants, and led us to hypothesize the impact of their distinct transcriptional signatures and their consequences for T-cell long-term persistence. Collectively, our findings establish immunoswitch receptors as a versatile and powerful tool for enhancing engineered T-cell therapies in the context of chronic viral infections and liver cancer, providing a rational and adaptable framework for overcoming inhibitory microenvironmental conditions while complementing existing antigen-specific CAR- and TCR-engineered T-cell products.

## 2 Materials and methods

### 2.1 Primary cells and cell lines

Human PBMC from healthy donors were isolated by Ficoll density gradient centrifugation and used for the transduction of PD-1-based immunoswitches, HBV-specific CARs, and TCRs. The study was approved by the local ethics committee, and blood donors provided written informed consent.

T cells used for retroviral transduction were either primary human T cells from PBMCs or human T cells expressing CARs and TCRs specific for the HBV S antigen (S-CAR 28z, TCR 4G or TCR WL12) (17, 33, 34). T cells were cultured in RPMI1640 supplemented with 1% L-glutamine, 1% non-essential amino acids, 1% sodium pyruvate, 1% penicillin/streptomycin, and 10% FCS (T cell medium, TCM). Human IL-2 (Peprotech, Thermo Fisher, Germany) was added at the concentration as indicated.

The used cell lines were the human hepatocellular carcinoma cell line HepG2 (HB-8065, ATCC), from which the HepG2-NTCP, HepG2.2.15, and HepG2-SML cell lines, with and without PD-L1 expression, were derived. Triple-parameter reporter (TPR) Jurkat cells (Jurkat TPR) were kindly provided by Prof. Peter Steinberg of the Institute of Immunology at the Medical University of Vienna, Austria (35). All HepG2-based cell lines were grown in collagen-coated culture flasks and plates using DMEM basic medium supplemented with 10% FCS at 37 °C and 5% CO_2_. Jurkat TPR cells were cultured using RMPI-basic with 10% FCS at 37 °C/5% CO_2_. Mycoplasma testing was performed weekly. All cell lines were confirmed to express the required molecules for the experiments by flow cytometry.

### 2.2 HBV-specific TCR functional avidity determination

Specific lysis of peptide-loaded T2 cells by transduced CD8+ and CD4+ T cells was determined by a chromium release assay. Target cells are labeled for 1 hour with sodium chromate^51^ (Hartmann GmbH), loaded with the corresponding peptide, and co-cultured with effector cells at defined E:T ratios for 4 hours. The radioactive chromium is released from the killed target cells into the co-culture medium. By measuring the radioactive intensity of the co-culture medium, the percentage of T cell-mediated killing capacity is calculated. The functional avidity of the T cells is calculated as the mean effective concentration (EC50) or half-maximal lysis using the killing values of T2 cells loaded with titrated peptide concentrations.

### 2.3 CARs, TCRs, and PD-1-based immunoswitch constructs and retroviral transduction

Three different PD-1-based immunoswitch constructs were designed. All of them formed a type I membrane protein structure, with the C-terminal portion corresponding to the PD-1 extracellular domain (ECD). While the PD-1_CD28 construct was linked to the transmembrane (TMD) plus intracellular domain (ICD) of CD28 (AA 153-220), PD-1_4-1BB and PD-1_OX40 constructs linked the PD-1 ECD to the 4-1BB (AA 214-255) and OX40 (AA 236-277) ICD using the PD-1 TMD. To support successful intracellular trafficking and surface expression, the PD-1 signal peptide (20 AA) was conserved across all three constructs. While the PD-1_CD28 construct and PD-1_4-1BB constructs and endogenous PD-L1 protein were kindly provided by Elfriede Noessner (31, 32), the PD-1_OX40 receptor was designed following the structural criteria of the PD-1_4-1BB receptor. The PD-1_OX40 sequence (ordered from GeneArt) was cloned into the pMP71 vector for retroviral transduction of primary T cells (CD3/CD28-activated PBMC), or HBV-specific T cells expressing the S-CAR 28z and its variations, as well as the TCR 4G or TCR WL12 (17, 33, 34). Retroviral transduction was performed as described (34). Briefly, human PBMC or HBV-specific engineered T cells were thawed and activated with 5 μg/mL of plate-bound OKT3 (provided by the Monoclonal Antibodies core facility, Helmholtz Munich, Germany) and 1 μg/mL of anti-CD28 (BD Bioscience) for 2 days in TCM with 100 U/mL IL-2. Thereafter, the T cells were split into four equal parts (1×10^6^ cells), each transduced with retroviral particles encoding the HBV-specific CAR or TCR and each one of the three PD-1 immunoswitch constructs. The control groups were transduced without immunoswitches, just expressing the corresponding CAR or TCR (mock–CAR/TCR cells) or no receptor at all (mock T cells). After 4 days, each transduced T cell condition was harvested and cultured for 12 days to facilitate expansion, with the IL-2 concentration adjusted to 100 U/mL every other day. Immunoswitch-transduced HBV-specific T cells were frozen on day 12 after transduction.

### 2.4 Membrane expression of immunoswitch receptors and target cell characterization

To analyze surface expression of PD-1-based immunoswitches, anti-PD1-APC (Invitrogen) was used in combination with anti-mouse TCRβ-constant region (mTCR)-FITC (BD Biosciences) to detect the TCR expression or Anti-human IgG-FITC (Sigma) to detect CAR expression, anti-CD4-PE (eBioscience), and anti-CD8-Pacific Blue (BioLegend). PD-L1 expression on HepG2 target cells was analyzed using anti-PD-L1-BV421 (Invitrogen). Near-IR fluorescent reactive dye (Thermo Fisher) was used for live/dead discrimination in all stainings. Flow cytometry and data analysis were performed with the Cytoflex S (Beckman Coulter) flow cytometer and FlowJo v10.10 software.

### 2.5 *In vitro* HBV infection experiments

For HBV infection, HepG2-NTCP cells were differentiated in 6-well plates using DMEM medium supplemented with 1% L-glutamine, 1% non-essential amino acids, 1% sodium pyruvate, 1% penicillin/streptomycin, and 10% FCS plus 2.5% DMSO (Differentiation medium). After 3 days of differentiation, the virus was added to 3 mL of HepG2 differentiation medium supplemented with 4% PEG 6000 (Merck). After 24 hours, the cells were washed 3 times with PBS and incubated in HepG2 differentiation medium until used for cytotoxicity assays.

### 2.6 CFSE staining

To determine cell proliferation, cells were stained using a carboxyfluorescein succinimidyl ester (CFSE) kit (Thermo Fisher). Cells were washed twice with PBS to remove serum, and 5–10 × 10^6^ cells/mL were stained with 1 μM CFSE in PBS (10 min, RT, in the dark). Following, five times the volume of TCM without IL-2 was added and incubated (5 min, on ice, in the dark). Cells were washed three times with TCM and then seeded on HBs antigen-precoated plates for stimulation. Dilution of CFSE was determined by flow cytometry after 96 hours of stimulation.

### 2.7 Jurkat TPR signaling assay

The Jurkat TPR cells were co-transduced to express the WL12 TCR and one of the three PD-1-based immunoswitch receptors (PD-1_CD28, PD-1_4-1BB, or PD-1_OX40) and subsequently sorted for double-positive expression of these molecules using the MA900 cell sorter (Sony). Jurkat TPR cells transduced with only the WL12 TCR were generated as a control reference, and untransduced cells were used as a negative control. Expression was determined by flow cytometry, using the anti-mouse TCRβ-constant region-FITC and anti-PD1-APC (Invitrogen) antibodies to stain the WL12 TCR. Near-IR fluorescent reactive dye (Thermo Fisher) was used for live/dead discrimination. Receptor-positive TPR-Jurkat cells were enriched by cell sorting and subsequently used for a stimulation experiment. Antigen-negative HepG2-NTCP cell line was used as the target cells. Cells were seeded in a collagen-coated plate and loaded with the corresponding HBs antigen-specific S_172–180_ peptide (exclusively recognized by the WL12 TCR) for 2 hours at 37 °C. After the loading step, peptide excess was carefully removed by washing with PBS, and the different Jurkat TPR-transduced and enriched cells were added in an effector-to-target (E:T) ratio of 1:1 and co-cultured for 24 hours. Unloaded target cells served as a negative co-culture control, as did unstimulated transduced Jurkat TPR cells. Expression of NFAT-GFP, NF-κB-CFP, and AP-1-mCherry reporter transcription factors was measured via flow cytometry after 24 hours of co-culture with the corresponding target cells.

### 2.8 Cytotoxicity assay, target cell co-cultures, and IFN-γ release assay

HepG-derived target cell lines (HepG2-NTCP, HepG2.2.15, and HepG2-SML) with or without PD-L1 surface expression were differentiated in cell culture flasks using DMEM differentiation medium for 3 days. Cells were then seeded onto 96-well electronic microtiter plates (ACEA Biosciences) at a density of 3×10^4^ cells/well. For co-culture assays, target cells were seeded at the desired concentration for the experimental setup. As for the effector T cells (TCR or CAR only and TCR or CAR plus PD-1 immunoswitches), transduced T cell numbers used for the assays were calculated based on the assessed surface expression (TCR or CAR single-positive cells, and TCR/CAR^+^ PD-1^+^ double-positive cells) to fulfill the 1:1 ratio condition. This adjustment allowed all co-culture conditions to contain similar amount of T cells expressing the relevant receptors (single TCR or CAR, and double-positive TCR or CAR plus PD-1 immunoswitches), minimizing surface expression variation observed after transduction.

Electrical impedance was measured every 30 minutes with an xCELLigence SP real-time cell analyzer (ACEA Biosciences) for 72 hours. Supernatants were collected from the electronic microtiter plates at the end of the 72-hour co-culture, and IFN-γ analysis was performed using a human IFN-γ uncoated ELISA kit (Thermo Fisher) according to the manufacturer´s instructions. Standard curves were fitted using the logistic-5PL regression type.

### 2.9 PD-1/PD-L1 blockage assay

HepG2-derived target cell lines (HepG2-NTCP and HepG2-SML) expressing surface PD-L1 were differentiated in 96-well electronic microtiter plates (ACEA Biosciences) at a density of 3×10^4^ cells/well for 3 days. Numbers of HBV-specific transduced T cells (CARs or TCRs) plus PD-1-based immunoswitch receptors were calculated according to TCR/CAR+ and PD-L1+ double positive expression percentages before being added to the co-culture at an E:T ratio of 1:1. Co-cultures were prepared using T-cells expressing the WL12 TCR with and without PD-1 immunoswitches, and with or without PD-1 and/or PD-L1 blocking antibodies (anti-PD-1 clone EH12.2H7, and anti-PD-L1 clone 29E.2A3 from Biolegend) at a concentration of 10μg/mL. Electrical impedance was measured every 30 minutes with an xCELLigence SP real-time cell analyzer (ACEA Biosciences) for 72 hours. Supernatants were collected from the electronic microtiter plates at the end of the 72-hour co-culture, and IFN-γ analysis was performed using a human IFN-γ uncoated ELISA kit (Thermo Fisher) according to the manufacturer´s instructions. Standard curves were fitted using the logistic-5PL regression type.

### 2.10 Repeated antigen rechallenge

HepG2-derived target cell lines (HepG2-NTCP, HepG2.2.15, and HepG2-SML) with or without PD-L1 surface expression were differentiated in cell culture flasks using DMEM differentiation medium for 3 days. Cells were then seeded onto 96-well electronic microtiter plates (ACEA Biosciences) at a density of 3 × 10^4^ cells/well. HBV-specific transduced T cells expressing the HBV-specific WL12 TCR plus each individual PD-1-based immunoswitch receptor were added at an E:T ratio of 1:1. T cells expressing only the WL12 TCR or no TCR alone were used as controls to evaluate the effect of the different immunoswitches. Electrical impedance was measured every 30 minutes with an xCELLigence SP real-time cell analyzer (ACEA Biosciences) for 72 hours. After the initial 72-hour co-culture, T cells were harvested from each well and transferred to a newly seeded target cell plate for a second round of co-culture. The same procedure was performed for a third and final round. After each co-culture round, killing capacity was analyzed using the normalized cell index data provided by the xCELLigence device. Supernatants were then collected from the electronic microtiter plates for IFN-γ secretion analysis, which was performed using a human IFN-γ uncoated ELISA kit (Thermo Fisher) according to the manufacturer´s instructions.

### 2.11 PD-L1 signal trans delivery assay

Evaluation of PD-L1 signal delivery in trans, uses WL12 TCR+ or S-CAR+ effector T expressing only the TCR or CAR, or in combination with either the PD-1_CD28 or the PD-1_4-1BB immunoswitches. Effector cells were then co-cultured with the HepG2 differentiated antigen expressing target cells HepG2.2.15 and HepG2-SML at a constant E:T ratio of 1:1. Antigen negative HepG2 cells with or without PD-L1 surface expression completed the co-culture set up as providers of membrane PD-L1 in different stimulator to effector ratios (E:S) 1:1, 1:0.2, 1:0.1, 1:0.05, 1:0.02 until 1:0; which decreases the amount of available stimulator cells stepwise. Supernatants from the different co-culture conditions were harvested after 72 hours, and IFN-γ was measured using the human IFN-γ uncoated ELISA kit (Thermo Fisher) according to the manufacturer´s instructions.

### 2.12 *In vivo* functionality of HBV-specific T cells carrying PD-1-based immunoswitches in a chronic HBV infection mouse model

Homozygous B6.129S7-Rag1^tm1Mom^ (Bl6.Rag 1^-/-^) mice were bred in-house in a specific pathogen-free animal facility. Persistent HBV replication was established by intravenous injection of 1.8×10^10^ genome equivalents of an adeno-associated virus vector containing a replication-competent 1.3-fold HBV genome (AAV-HBV) and 1.5×10^11^ genome equivalents of an adeno-associated virus vector expressing the human histocompatibility surface molecule HLA-A*02:01 (AAV-HLA-A2) (36). T cells were isolated from CD45.1^+/+^ C57BL/6 donor mice and retrovirally transduced with the HBV-specific 4G TCR alone or in combination with the PD-1_CD28 or PD-1-4-1BB immunoswitch constructs as described previously. 6 weeks after AAV-HBV infection, 0.2x10^6^ TCR/immunoswitch- or mock-transduced T cells were transferred by i.p. injection into each mouse. HBsAg, HBeAg, and ALT levels were quantified as described previously (37).

### 2.13 Cytokine secretion and exhaustion phenotype evaluation of mouse cells from spleen and liver

Liver-associated lymphocytes were isolated as described previously (37). For intracellular cytokine staining, cells were stimulated for 16h in the presence of 1 μg/mL Brefeldin A (Sigma-Aldrich) with a 100nM concentration of the HBs antigen-specific peptide S_20-28_ (exclusively recognized by 4G TCR when loaded into HLA-A*02:01 surface molecules). Surface staining was performed using anti-CD45.1-BV650 (BD), anti-TCRVb5.1-FITC (Beckman Coulter), anti-PD-1-PerCPeF710 (eBioscience), and anti-CD8-PB (BD) antibodies. Near-IR fluorescent reactive dye (Thermo Fisher) was used for live/dead discrimination in all stainings. Intracellular cytokine staining was performed using a Fixation/Permeabilization kit (BD) according to the manufacturer’s instructions, with anti-IFN-γ-APC (eBioscience), anti-TNF-α-PE-Cy7 (BD), and anti-IL-2-PE Dazzle 594 (BioLegend) antibodies. For the immunophenotype panel staining, surface staining of splenocytes and liver-associated lymphocytes was performed using CD45.1-APC (eBioscience), anti-TCRVb5.1-FITC (Beckman Coulter), anti-PD-1-PerCPeF710 (eBioscience), anti-CD8-PB (BD), anti-Lag-3-PE Dazzle 594 (BioLegend), and anti-4-1BB-PECy7 antibodies. Near-IR fluorescent reactive dye (Thermo Fisher) was used for live/dead discrimination. Intracellular TOX staining was performed using a FOXP3/Transcription Factor staining buffer set 1 kit (eBioscience) according to the manufacturer’s instructions with anti-TOX-PE (Invitrogen). 10 μL of Count Bright™ Absolute counting beads was added at the end of the staining to calculate the absolute cell numbers present in the liver (Invitrogen) according to the manufacturer´s instructions. Cells were analyzed using a CytoFLEX S (Beckman Coulter), and data were analyzed with FlowJo 10.4 software.

### 2.14 HBV DNA quantification

HBV-DNA was quantified in DNA extracted from liver tissue by real-time polymerase chain reaction (PCR). The Nucleospin Tissue Kit was used for total cellular DNA extraction (Macherey & Nagel, Düren, Germany) according to the manufacturer’s instructions. HBV DNA (primers: HBV1745 GTTGCCCGTTTGTCCTCTAATTC; HBV1844 GGAGGGATACATAGAGGTTCC-TTGA) and murine prion protein PrP (primers: mPrP_fwd GCGGTACATGTTTTCACGGTAGTA; mPrP_rev GAGCAGGCCCATGATCCA) as a reference gene were amplified by qPCR on a LightCycler 480 system using SYBR Green I Master (Roche, Mannheim, Germany). Cycling conditions for HBV primers: 2 minutes 50 °C, 10 minutes 95 °C, followed by 45x (15 seconds 95 °C, 1 minute 60 °C). Cycling conditions for reference mPrp: 2 minutes 50 °C, 10 minutes 95 °C, followed by 45x (15 seconds 95 °C, 1 minute 60 °C).

### 2.15 IL-6 measurement

Mice were bled at different time points after T-cell therapy treatment, and plasma samples from days 21, 35, and 58 were used for IL-6 measurement, which was performed using a mouse IL-6 uncoated ELISA kit (Invitrogen - Thermo Fisher) according to the manufacturer´s instructions.

### 2.16 Histology and immunohistochemistry

Liver, kidney, and heart tissue samples were fixed in 4% buffered formalin for 24h and were paraffin-embedded. Tissue sections were then prepared using a rotary microtome (HM355S, Thermo Fisher). Immunohistochemistry was performed using a Bond RX system (Leica) with the anti-HBcAg primary antibody (LSBio, LS-C312204, 1:50 dilution) and a horseradish peroxidase-coupled secondary antibody. Briefly, the slides were deparaffinized using a deparaffinization solution pre-treated with epitope retrieval solution 2 (corresponding to EDTA buffer, pH 9) for 40 minutes. Antibody binding was detected with a polymer refine detection kit without a post-primary reagent, and the reaction was visualized as a dark brown precipitate with DAB. Counterstaining was done with Hematoxylin-Eosin. Slides were scanned using an Aperio AT2 slide scanner (Leica). HBcAg-positive hepatocytes were determined based on the localization, intensity, and distribution of the signal in 10 random view fields (8x magnification). The mean numbers of HBcAg-positive hepatocytes were quantified per mm². An experienced liver pathologist evaluated histopathologic changes and rated the severity of inflammation and T cell infiltration in liver tissue.

### 2.17 Statistical analysis

Statistical analyses were performed using GraphPad Prism 10.6.1 software. Statistical differences were calculated using a 2-way analysis of variance (ANOVA) with Dunnett’s multiple-comparison correction.

## 3 Results

### 3.1 An additional co-stimulatory domain in the 2^nd^ generation HBV-specific S-CAR 28z structure fails to enhance the killing of HBV-infected HepG2-NTCP cells.

**Figure 1:**
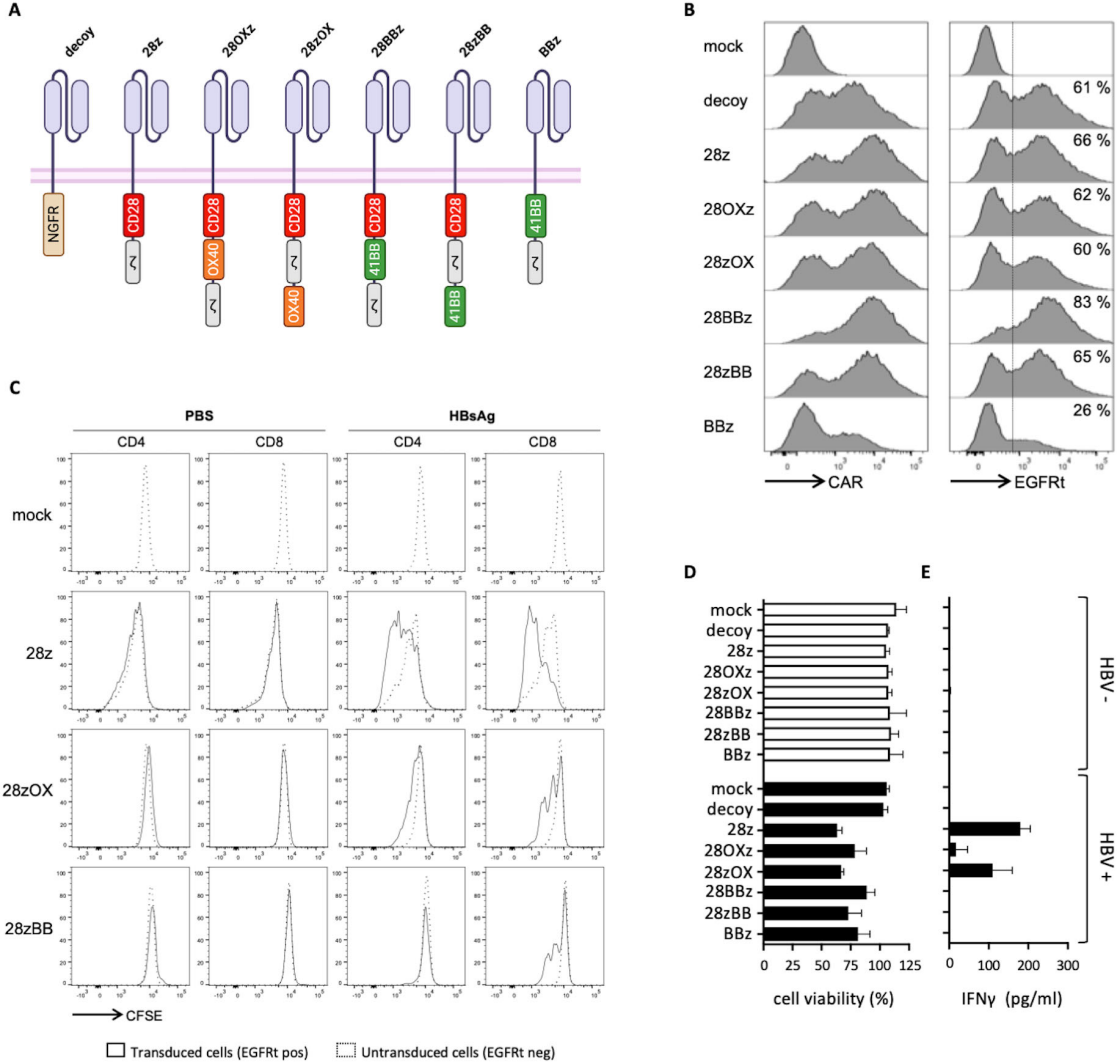
Additional co-stimulatory sequences do not improve the 2^nd^-generation CAR 28z. Human PBMC were retrovirally transduced to express different 2^nd^- and 3^rd^-generation S-CAR constructs. **(A)** Scheme of different S-CAR constructs. All versions carry the C8 scFv (light purple). S-CARs contain a wild-type IgG1 spacer consisting of hinge-CH2-CH3 domains. Utilized intracellular domains were derived from nerve growth factor receptor (NGFR, light brown), CD28 (red), OX40 (orange), 4-1BB (green), and CD3ζ (light grey). Created in BioRender. Olguin, L. (2026) https://BioRender.com/5sc9fga **(B)** Histograms of flow cytometry analysis that determine transduction rate via EGFRt (right panel), and S-CAR surface expression (left panel). Retrovirally transduced PBMC were cultured with HBV-infected (MOI 100) HepG2-NTCP cells (E:T=1:1). Cytotoxicity and activation effects induced by different S-CAR constructs were compared. **(C)** CFSE-labeled CAR-transduced PBMC were cultured on HBsAg-coated wells (5μg/mL) or PBS control wells. Dilution of CFSE was determined by flow cytometry after 96 hours. Cells were pre-gated on CD4^+^ and CD8^+^ T cells, and on transduced (EGFRt positive, solid lines) and untransduced (EGFRt negative, dotted lines) cells. **(D)** Cytotoxicity is depicted as the normalized cell index from xCELLigence RTCA (normalized to the start of co-culture) at 80 hours of co-culture. **(E)** Secreted IFN-γ in supernatant after 80 hours. Shown are the mean values of triplicates from one representative experiment repeated 3 times.

The S-CAR used in previous studies (17, 33) incorporated the signaling domains of CD28 and CD3ζ (28z). To evaluate the additive effect of extra signaling domains, the 2^nd^-generation S-CAR was modified by adding OX40 (OX) or 4-1BB (BB) intracellular domains at the C-terminal of CD3ζ (z) or between the CD28 (28) intracellular domain and CD3ζ. With these modifications, four 3^rd^-generation CARs were generated, designated as 28OXz, 28zOX, 28BBz, and 28zBB, based on the corresponding positions of the added domains within the CAR intracellular domain. An additional second-generation S-CAR, which contains a single 4-1BB domain instead of the CD28 domain (BBz), was also included. A decoy S-CAR containing an intracellular domain of the nerve growth factor receptor (NGFR) served as a negative control (**Figure 1A**). All constructs expressed an additional intracellularly truncated epidermal growth factor receptor (EGFRt) (38) to monitor CAR expression on PBMC-derived T cells by flow cytometry. The transduction rate, determined by EGFRt expression, was between 60% to 66% for most constructs. Only the 28BBz S-CAR displayed higher membrane expression (83%), while the 2^nd^ generation BBz S-CAR had lower expression levels (26%) (**Figure 1B**).

The proliferation of activated T cells is essential for increasing the number of effector cells that contribute to the antiviral effect. For this reason, we compared T-cell proliferation upon T-cell stimulation among the different S-CAR variants using a carboxyfluorescein succinimidyl ester (CFSE) dilution assay. Flow cytometry analysis revealed that CD4+ and CD8+ S-CAR T cells specifically proliferated upon contact with HBsAg, compared to control cells (PBS-treated or mock-transduced/EGFRt-negative T cells). 2^nd^ generation 28z as well as 3^rd^-generation S-CAR 24zOX responded to HBsAg stimulation with proliferation of both CD4+ and CD8+ T-cell subsets, while 28zBB showed proliferative induction only in the CD8+ T cells (**Figure 1C**). Overall, the proliferative capacity of 2^nd^-generation CAR-T cells was not augmented by the addition of further co-stimulatory signaling domains.

To mimic the relatively low-density expression of the S protein on HBV-infected cells, HepG2-NTCP cells were infected with HBV. We compared the potency of the different S-CAR variants in activating T cells to secrete IFN-γ and kill HBV-infected cells in co-culture experiments. T cells expressing S-CARs with only CD28 or an additional co-stimulatory domain, OX40, showed a similar decrease in target cell viability. However, the functionality of the 2^nd^-generation S-CAR 28z was not increased by the addition of the OX40 signaling domain. In contrast, all S-CARs that contained a 4-1BB domain exhibited lower cytotoxicity (**Figure 1D**). A similar pattern was observed when supernatants were analyzed for secreted IFN-γ, indicating T-cell activation (**Figure 1E**). Only S-CAR T cells that did not contain the 4-1BB signaling domain secreted IFN-γ. Collectively, the results did not provide evidence for an advantage of adding OX40 or 4-1BB signaling domains into the 28z S-CAR; rather, negative effects were observed with additional 4-1BB or OX40 domains.

### 3.2 Spatially separated co-stimulation by a PD-1 immunoswitch receptor improves S-CAR 28z functionality

**Figure 2:**
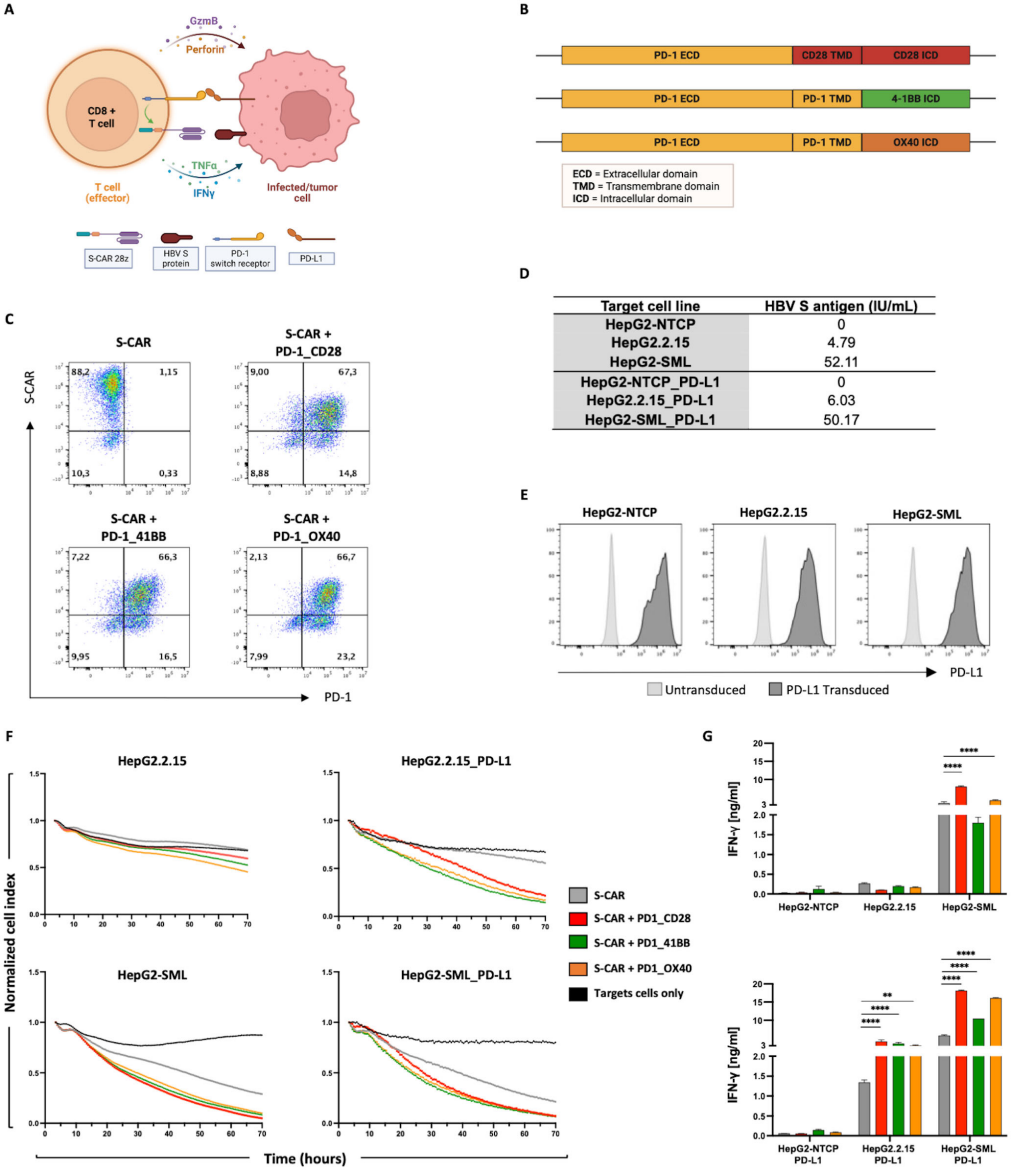
Spatially separated immunoswitch-driven co-stimulation improves CAR 28z functionality. **(A)** Graphic scheme depicting CAR T cell/Target cell interaction with spatially separated immunoswitch delivered co-stimulation. Created in BioRender. Olguin, L. (2026) https://BioRender.com/f8vdl0t **(B)** Structural representation of the three PD-1-based immunoswitches with CD28 (red), 4-1BB (green), and OX40 (orange) signaling domains. **(C)** Human PBMC were retrovirally transduced simultaneously to express HBV-specific S-CAR 28z and the three corresponding PD-1 switch receptors. Surface expression was measured by flow cytometry 13 days after transduction. The percentage of double-positive cells within the gated live, single, CD3+, CD8+ population is displayed as pseudo color dot plots. S-CAR Mock transduced T cells were used as a negative control. **(D)** HBs Antigen concentration on untransduced and PD-L1 transduced target cells was measured after 3 days of target cell differentiation. HBs Antigen was measured using the ARCHITECT device, and concentrations are shown in the table as IU/mL. **(E)** HepG2-based target cells expressing no, low, and high amounts of HBV S antigen (NTCP, 2.15, and SML) were used for PD-L1 retroviral transduction. PD-L1 transduced target cells were enriched by FACS, and the percentage of PD-L1 expression is shown in the histograms (dark grey). Untransduced target cells were used as a negative control (light grey). **(F)** S-CAR 28z expressing T cells containing the three different PD-1-based immunoswitches were co-cultured for 72 hours with each corresponding differentiated target cell line with or without PD-L1. Supernatant was collected after 72 hours. Graphs show target cell killing with and without PD-L1 expression over time using the xCELLigence real-time cell analyzer. Lines depicted in the graphs representing the mean of triplicate samples correspond to S-CAR 28z T cells co-expressing the PD-1_CD28 (red), PD-1_4-1BB (green), or PD-1_OX40 (orange) immunoswitch. All graphs show S-CAR 28z expressing T cells without immunoswitches (grey) and target cells only (black) as controls. **(G)** IFN-γ was measured in supernatants of the different cytotoxicity co-culture plates after 72 hours. Color code of the bar graphs follows the same as for the cytotoxicity graphs. HepG2-NTCP target cells were used as an unspecific activation control. Shown are the mean values of triplicates from one representative experiment repeated 3 times. Error bars are the standard deviation. Statistical analysis was performed using a 2-way analysis of variance (ANOVA) with Dunnett’s multiple-comparison correction. *P-*values represented as follows: 0.0021 (**), and <0.0001 (****).

Because adding co-signaling domains to the 2^nd^-generation HBV-specific S-CAR 28z failed to improve its functional capacity, we decided to deliver co-stimulation via a separate molecule. We chose the previously described PD-1-based immunoswitch receptor as a basis (31, 32). As depicted in **Figure 2A**, using immunoswitch receptors, the two key immunological signals involved in T cell activation mimic the physiologic topological distribution, unlike the 3^rd^-generation CAR concept we explored before.

The immunoswitch receptors designed contained the PD-1 extracellular domain fused to the signaling domains of CD28, 4-1BB, or OX40 (**Figure 2B**). All variants were successfully co-expressed with HBV-specific S-CAR 28z T cells, with double-positive expression levels of ~66% across all three immunoswitches (**Figure 2C**). The modular delivery of extra co-stimulatory signals through the immunoswitches was tested in co-culture assays using hepatoma target cells HepG2 expressing low (HepG2.2.15) or high (HepG2-SML) levels of HBV S antigen, as well as HBV-antigen negative target cells (HepG2-NTCP) (**Figure 2D**). The same target cells were successfully transduced to express the PD-L1 inhibitory ligand and were enriched by FACS sorting (**Figure 2E**), without altering antigen levels (**Figure 2D**). These target cells were used to test the functional quality of the S-CAR T cells that expressed additional immunoswitch receptors (**Figures 2F, G**).

S-CAR-T cells that expressed either one of the three immunoswitches exhibited enhanced killing of HepG2.2.15 (low antigen level) if the HepG2.2.15 additionally expressed PD-L1 (**Figure 2F**, right panels). Improving cytotoxicity of S-CAR T cells by the immunoswitches was more evident in the low antigen-expressing HepG2.2.15 target cells (top panels) compared to the HepG2-SML with high antigen levels (bottom panels), as these cells were already efficiently killed by the S-CAR 28z T cells. An improved killing activity was only present when effector S-CAR cells recognized the antigen expressed on the surface of PD-L1+ target cells, while the activation was less evident in the absence of PD-L1 (**Figure 2F**).

The effect of the immunoswitches was also reflected in the capacity of the S-CAR cells to secrete IFN-γ when co-cultured with target cells with and without PD-L1 expression. S-CAR 28z-T cells expressing an additional immunoswitch receptor surpassed the activity of S-CAR T cells only when interacting with PD-L1-positive target cells (**Figure 2G**). Comparing the different immunoswitches, the PD-1_CD28 immunoswitch most strongly co-activated IFN-γ secretion, followed by the other two receptors containing the 4-1BB and OX40 signaling domains. Moreover, if the target cell expressed the antigen at a low level (HepG2.2.15), high PD-L1 expression on the target cells and co-expression of immunoswitches and the S-CAR were required to activate the T cells to secrete IFN-γ. These data indicated that the activation of S-CAR T cells could be improved by an immunoswitch receptor that provided additional co-stimulation upon PD-1/PD-L1 interaction.

### 3.3 Expression and functional characteristics of PD-1 immunoswitches on HBV-specific TCR T cells

**Figure 3:**
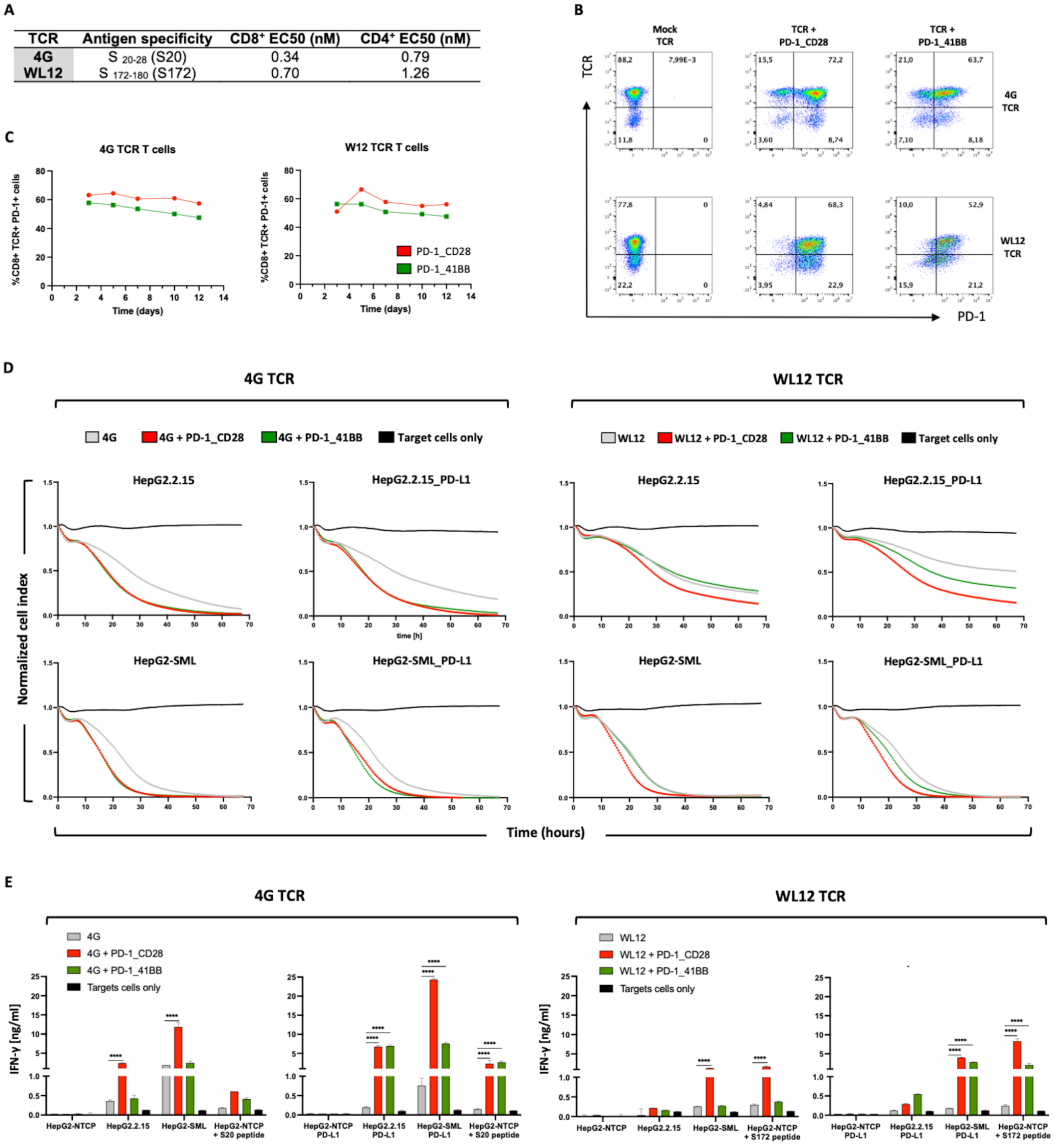
Characterization of PD-1 immunoswitches using HBV-specific TCR T cells. Human primary T cells were retrovirally transduced with the 4G TCR or WL12 TCR and additionally co-transduced with either the PD-1_CD28 or PD-1_4-1BB immunoswitch receptors. Transduced cells were co-cultured for 72 hours with differentiated low and high-expressing HBs antigen target cell lines with or without PD-L1. Supernatants were collected at the end point of the killing assay. **(A)** EC50 values for specific lysis by HBV-specific T-cell receptors after transduction into CD8+ or CD4+ T cells. Mean effective concentration (EC50) describes the peptide concentration in nM at which half of the target cells are specifically lysed. Using GraphPad Prism, non-linear regression curves were fit to the raw data, and EC50 values were derived from them. **(B)** Surface expression was measured by flow cytometry at 3, 5, 7, and 12 days after transduction using a murine TCR beta chain antibody for the TCR staining and a PD-1 antibody for the immunoswitch receptors. The percentage of double-positive cells within gated live, single, CD8+ populations is displayed in the upper right quadrant of the dot plots. Mock-transduced T cells were used as a negative control. **(C)** Percentage of PD-1 positive cells on CD8+ TCR+ cells at the different time points after transduction. TCR+ T cells transduced with PD-1_CD28 immunoswitch are depicted in red, and TCR+ T cells transduced with the PD-1_4-1BB immunoswitch are depicted in green. The left graph shows expression kinetics on 4G TCR T cells, and the right graph shows expression kinetics on WL12 TCR T cells. Also, transduced cells were co-cultured with differentiated low- and high-expressing HBs antigen target cell lines (HepG2.2.15 and HepG2-SML) for 72 hours, with or without PD-L1. Supernatants were collected at the end point of the killing assay. **(D)** Graphs showing killing of target cells (HepG2.2.15 and HepG2-SML) with and without PD-L1 expression over time using the xCELLigence real-time cell analyzer. Graphs on the left correspond to the T cells expressing the 4G TCR, while the graphs on the right correspond to the activity of WL12 TCR T cells. Graphs depict TCR-expressing T cells without immunoswitches (light grey), T cells expressing the TCR plus PD-1_CD28 (red), and PD-1_4-1BB (green). Target cells only (black) were used as controls. **(E)** IFN-γ was measured in supernatants of the different cytotoxicity co-culture plates after 72 hours using the human IFN-γ ELISA kit. Peptide-loaded HepG2-NTCP cells were used as a positive control. Shown are the mean values of triplicates from one representative experiment repeated 3 times. Error bars are the standard deviation. Statistical analysis was performed using a 2-way analysis of variance (ANOVA) with Dunnett’s multiple-comparison correction. *P-*values represented as follows: <0.0001 (****).

The observation that HBV-specific S-CAR 28z killing and IFN-γ secretion were improved when T cells co-expressed PD-1-based immunoswitches encouraged us to analyze whether similar effects would occur in the context of TCR-T cells. HBV-specific TCRs were selected that bound their cognate HBV S-peptide with high-avidity (TCR 4G; EC50 0.34 for CD8 T and 0.79 for CD4 T) or low-avidity (TCR WL12, EC50 0.7 for CD8 T and 1.26 for CD4 T), respectively (**Figure 3A**), determined using peptide titration and target cell killing of T2 cells.

4G-TCR and WL12-TCR T cells were co-transduced with PD-1_CD28 and PD-1_4-1BB immunoswitches. Co-expression of the TCRs and PD-1_CD28 or PD-1_4-1BB immunoswitches was confirmed by flow cytometry (**Figure 3B**). The frequencies of PD-1_CD28 immunoswitch expressing T cells were slightly higher (between 68 – 72%) than those co-expressing PD-1_4-1BB immunoswitch (between 52 – 63%). Co-expression proved stable over a 13-day period of T cell expansion (**Figure 3C**). PBMCs that followed the same transduction protocol but without the immunoswitch expression vector were used as a negative control (mock T cells).

Next, we evaluated the modular delivery of co-stimulatory signals through the immunoswitches on our TCR-T cells using co-culture assays with hepatoma target cells HepG2 expressing low (HepG2.2.15) or high (HepG2-SML) levels of HBV S antigen, as well as antigen-negative target cells (HepG2-NTCP) - all three with or without PD-L1 surface expression. Killing capacity was improved for both high- and low-avidity HBV-specific T cell receptors if co-stimulation was provided through immunoswitches (**Figure 3D**). High-avidity 4G TCR-T cells effectively killed antigen-positive target cells regardless of antigen expression levels or PD-L1 expression. Immunoswitches improved killing by 4G-TCR T cells with no clear differences between PD-1_CD28- and PD-1_4-1BB (**Figure 3D**, left panels). WL12-TCR T cells showed a similar killing pattern when co-cultured with high-antigen-expressing target cells that did not express PD-L1. On PD-L1 overexpressing cells and on low-antigen expressing cells, WL12-TCR-T cells carrying the PD-1_CD28 immunoswitch receptor showed stronger killing than those expressing PD-1_4-1BB (**Figure 3D**, right panels).

IFN-γ secretion was analyzed 72 hours after the initiation of the co-cultures. IFN-γ levels indicated that T cells expressing either the 4G TCR or the WL12 TCR secreted more cytokines when the T cells co-expressed the PD-1-based immunoswitches and were co-cultured with PD-L1-positive target cells (**Figure 3E**). Cells expressing the PD-1_CD28 receptor produced more IFN-γ, even on PD-L1-negative target cells. Moreover, in combination with the immunoswitches, the antigen recognition threshold of the low-avidity TCR WL12 was reduced, as observed in co-cultures with low-antigen-expressing HepG2.2.15 target cells.

**Figure 4:**
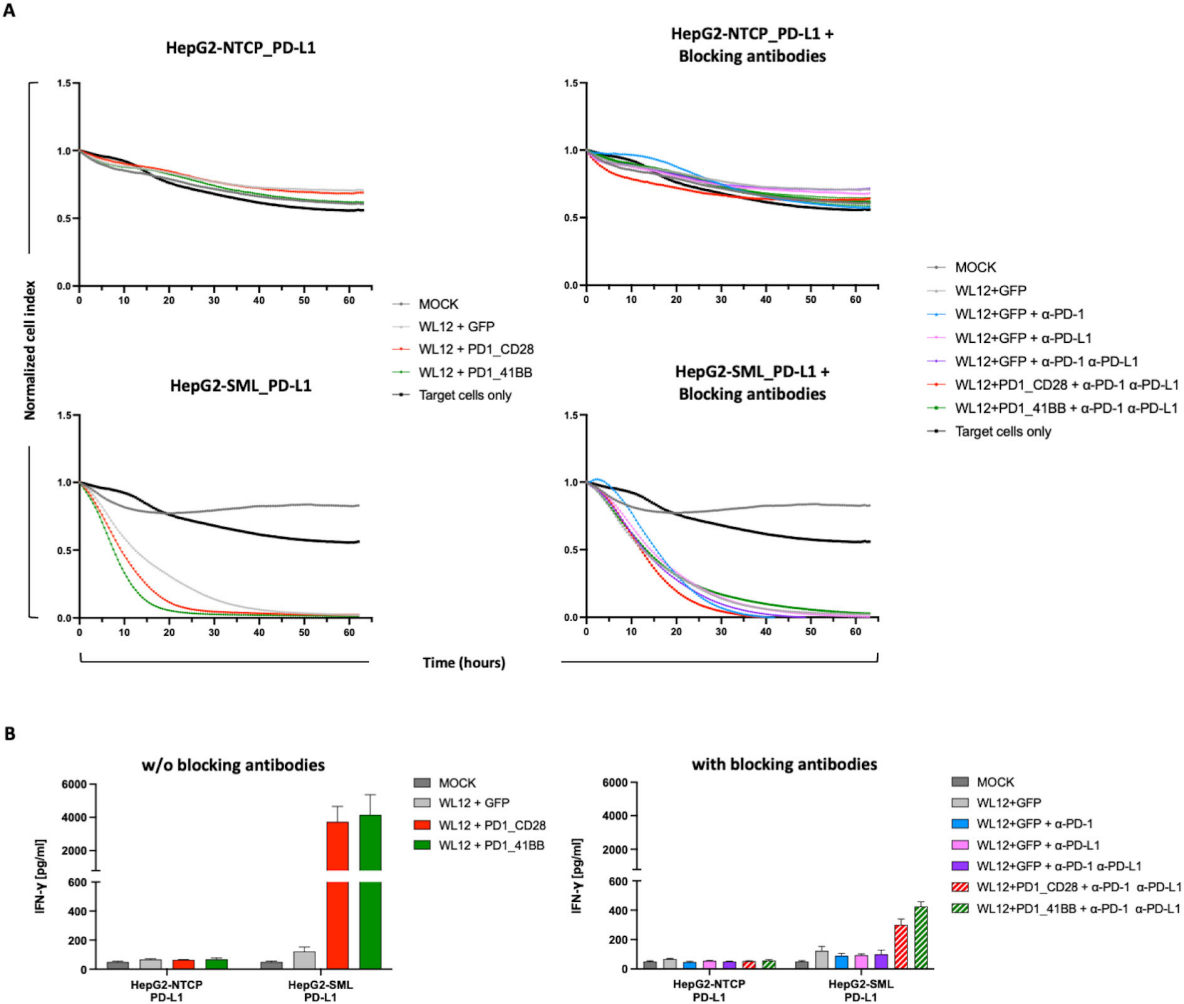
Effect of PD-1/PD-L1 blockage on HBV-specific T cells alone or in combination with PD-1-based immunoswitches. Human primary T cells were retrovirally transduced with the WL12 TCR and additionally co-transduced with either the PD-1_CD28 or PD-1_4-1BB immunoswitch receptors. Transduced cells were co-cultured for 72 hours with differentiated antigen-negative and high-expressing HBs antigen target cell lines with or without PD-L1. Supernatants were collected at the end point of the killing assay. **(A)** Graphs showing killing of PD-L1 expression target cells (HepG2-NTCP and HepG2-SML) over time using the xCELLigence real-time cell analyzer. Graphs on the left correspond to the co-culture activity of WL12 TCR T cells with and without PD-1 immunoswitches, and without added PD-1 and/or PD-L1 blocking antibodies. Graphs on the right show the activity of transduced T cells co-cultured with 10 μg/mL of PD-1 and/or PD-L1 blocking antibodies. Target cells only (black) and untransduced Mock cells (dark grey) were used as controls. **(B)** IFN-γ was measured in supernatants of the different co-cultures after 72 hours using the human IFN-γ ELISA kit. Shown are the mean values of triplicates from one representative experiment repeated 2 times.

Immunoswitch receptors increased the activation threshold and killing capacity, particularly in low-avidity TCR-T cells, in the presence of PD-L1. When additional blocking antibodies directed against PD-1 and/or PD-L1 were used, the killing capacity and IFN-γ secretion of T cells carrying the immunoswitches were suppressed, confirming that the enhancing effect was driven by the interaction of the immunoswitches with PD-L1 expressed on target cells. The addition of blocking antibodies did not affect the activity of WL12 HBV-specific TCRs lacking PD-1 immunoswitches, consistent with the observation that WL12 transgenic T cells do not express endogenous PD-1 (**Figure 4A** and **B**).

### 3.4 Signaling characterization of PD-1 immunoswitches using triple parameter reporter (TPR) Jurkat cells

**Figure 5:**
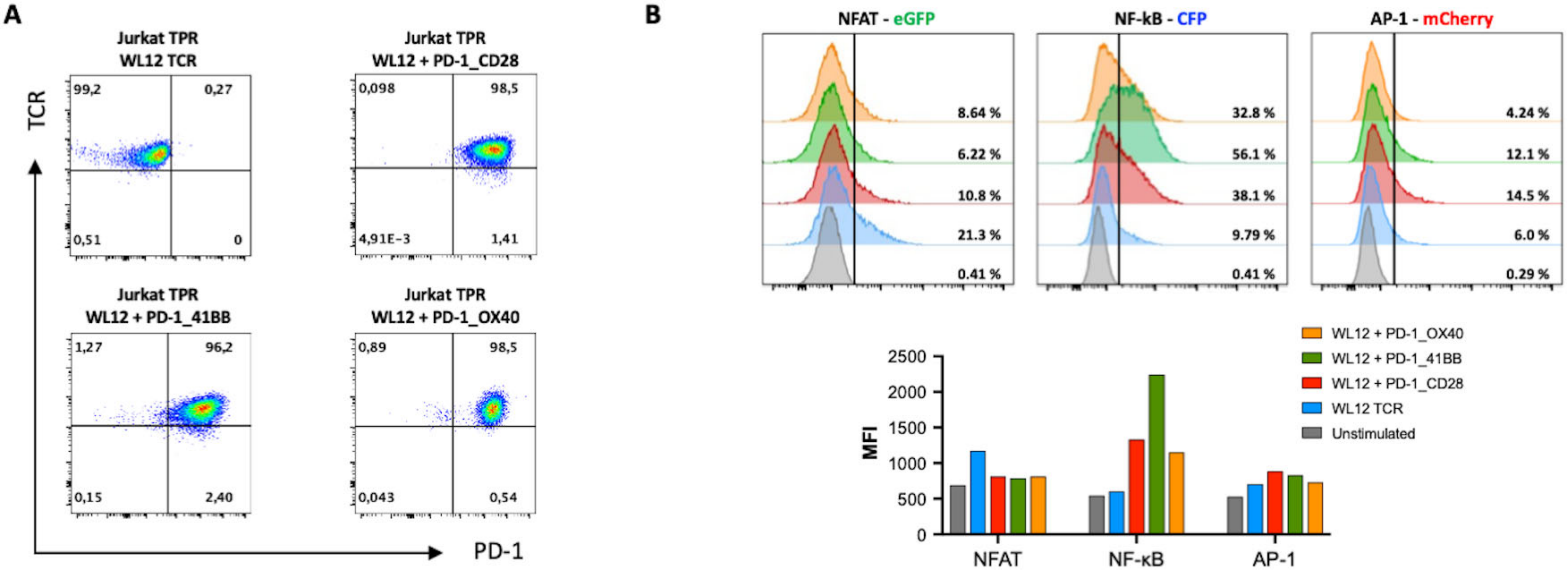
Signaling characterization of PD-1 immunoswitches using triple parameter reporter Jurkat cells (Jurkat TPR). Jurkat TPR reporter cells were retrovirally transduced with the WL12 TCR and, in addition, with the PD-1_CD28, PD-1_4-1BB, and PD-1_OX40 immunoswitch receptors, followed by double-positive (TCR+ PD-1+) cell sorting enrichment. **(A)** Dot plots showing the expression analysis of the PD-1-based immunoswitch receptors (x axis) and HBV-specific WL12 TCR (y axis) after sorting enrichment on the surface of Jurkat TPR cells using flow cytometry. **(B)** Enriched Jurkat TPR cells were co-cultured for 24 hours with peptide-loaded HepG2-NTCP cells to evaluate the additive effect of the PD-1 immunoswitches when co-expressed with the WL12 TCR. The depicted histograms show NFAT-GFP (left panel), NF-κB-CFP (center panel), and AP-1-mCherry (right panel) activation. The graph shows corresponding MFI values after 24 hours of co-culture-induced activation. Results correspond to one representative experiment of three repeats. Histograms and bars colors correspond to unstimulated Jurkat TPR cells (grey), WL12 TCR (blue), WL12 + PD-1_CD28 (red), WL12 + PD-1_4-1BB (green), and WL12 + PD-1_OX40 (orange) transduced Jurkat TPR cells.

To characterize the signaling effects of the PD-1-based immunoswitches in the context of HBV-specific TCRs, the low-affinity HBV S-specific WL12 TCR and the immunoswitches PD-1 immunoswitches with the CD28, 41BB, and OX40 signaling domains were transduced into a triple-parameter reporter Jurkat cell line (Jurkat TPR) (**Figure 5A**). The Jurkat TPR reporter cells allow simultaneous assessment of the activity of transcription factors that play key roles in T cell activation by detecting NF-κB via cyan fluorescent protein (CFP), NFAT via enhanced green fluorescent protein (EGFP), and AP-1 via mCherry.

TCR signaling was initiated by co-culturing Jurkat TPR cells with peptide-loaded HepG2-NTCP target cells. The control WL12 TCR-transduced Jurkat cells displayed 21.3% NFAT-active cells, 9.79% NF-κB-active cells, and 6% AP-1-active cells, measured after 24 h. All immunoswitches increased NF-κB. AP-1 signal intensity was minimally increased in PD-1_CD28- and PD-1_41BB- transduced Jurkat TPRs. All immunoswitches reduced NFAT activation compared with Jurkat cells expressing only the WL12 TCR (**Figure 5B**). PD-1_41BB improved NF-κB signaling and decreased NFAT signaling most strongly. The PD-1_OX40 immunoswitch primarily activated the NF-kB pathway, with minimal activity in NFAT and AP-1. The results were comparable when mean fluorescence intensity (MFI) was used instead of frequency (**Figure 5B**, bar graph).

### 3.5 *In vivo* activity of HBV-specific TCR engineered T cells co-expressing PD-1-based immunoswitches

**Figure 6:**
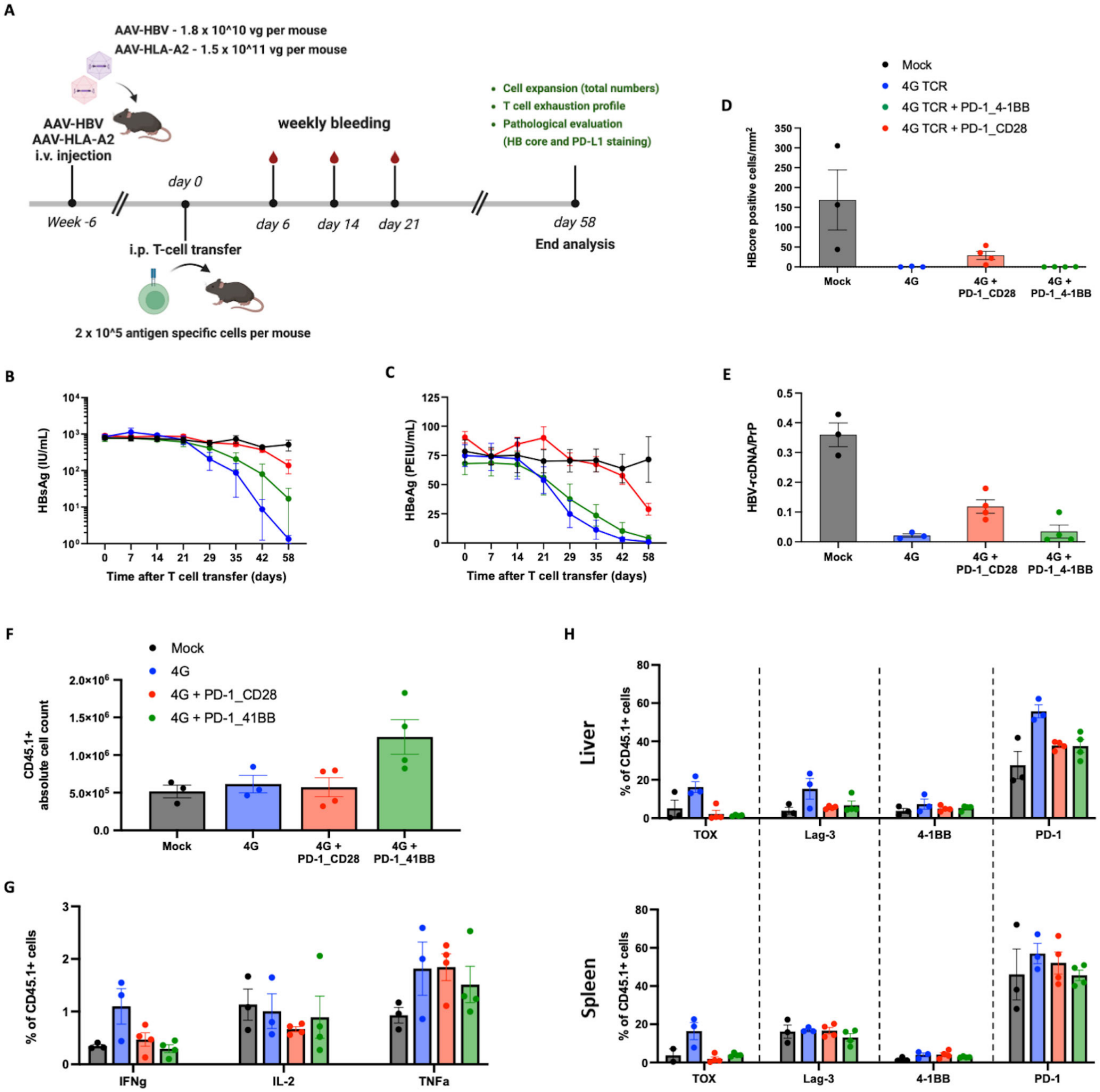
*In vivo* functionality of PD-1-based immunoswitches in combination with HBV-specific TCR T cells. **(A)** Experimental timeline. Persistent HBV replication and HLA-A*0201 expression were established in Bl6.Rag1-/- mice using AAV-HBV/AAV-HLA-A2. 0.2x10^6^ CD45.1^+/+^ syngeneic T cells expressing the 4G TCR alone or in co-expression with murine PD-1_CD28 or PD-1_4-1BB immunoswitches were transferred into each mouse at week 6 after AAV-HBV/AAV-HLA-A2 infection (n=5 per group). 4 animals served as mock controls. End-point analyses were performed at day 58 as indicated. Created in BioRender. Olguin, L. (2026) https://BioRender.com/p5yla73. Kinetics of **(B)** HBsAg, and **(C)** HBeAg levels in serum. **(D)** Quantification of HBV-DNA levels from liver tissue relative to the murine prion protein (PrP). **(E)** Graph depicting the number of HBcAg+ hepatocytes per square millimeter, as determined by quantitative analysis of immunohistochemical staining. **(F)** Absolute count of transferred CD45.1+ T cells in the liver per treatment group. Calculated using Count Bright™ Absolute counting beads (Invitrogen) according to the manufacturer´s instructions. **(G)** Frequencies of IFN-γ-, IL-2-, and TNF-α-positive transferred T cells among gated CD45.1+ T cells isolated from liver detected by intracellular cytokine staining after 16 hours of *ex vivo* stimulation with 100nM specific peptide. **(H)** Frequencies of CD45.1+ transferred T cells expressing TOX, Lag-3, 4-1BB, or PD-1 were detected by flow cytometry staining of T cells isolated from the liver and spleen. Symbols and lines represent individual mice or the mean values of treatment groups for HBsAg and HBeAg.

To translate our observations in cell culture into a physiological setting, HBV-specific TCR-T cells (either expressing the TCR alone or co-expressing murine PD-1_CD28 or PD-1_4-1BB immunoswitches) were evaluated in chronic HBV carrier Rag1^-/-^ mice (**Figure 6A**). To establish HBV replication in the liver, the mice were infected with an AAV vector that transduced the liver with a replication-competent HBV genome and the human HLA-A2 allele, allowing presentation of the cognate peptide of our TCR on hepatocytes. 6 weeks after AAV-HBV infection, the mice received 2×10^5^ mouse T cells expressing the 4G TCR, with or without PD-1_CD28 or PD-1_4-1BB immunoswitches.

Serum levels of viral HBsAg and HBeAg dropped over time for the groups of TCR-expressing T cells only and TCR T cells expressing 4G plus PD-1_4-1BB (**Figures 6B**, **C**). Immunohistochemical staining of liver tissue showed low numbers of HBc antigen-expressing cells, corresponding to low viral DNA levels, in groups with low HBsAg and HBeAg levels (**Figures 6D**, **E**). Consistent with their enhanced performance *in vitro*, T cells expressing PD-1_4-1BB exhibited superior persistence *in vivo*, as reflected by higher numbers of adoptively transferred T cells recovered from the liver at endpoint compared to the 4G TCR-only cells (**Figure 6F**). Following S peptide-specific ex vivo restimulation, liver-isolated lymphocytes showed increased IFN-γ levels only in the 4G TCR without immunoswitch group, while TNF-α levels were increased across all groups compared to mock T cells. No differences were detected for IL-2 secretion (**Figure 6G**).

Although antigen clearance kinetics were broadly similar between TCR-only and PD-1_4-1BB–modified T cells, the immunoswitch-equipped TCR-T cells showed lower expression of the exhaustion-associated transcription factor TOX in T cells isolated from liver and spleen. Similar to TOX, elevated expression of Lag-3 and PD-1 exhaustion markers was observed mainly in T cells isolated from the liver of the TCR-only group (**Figure 6H**).

**Figure 7:**
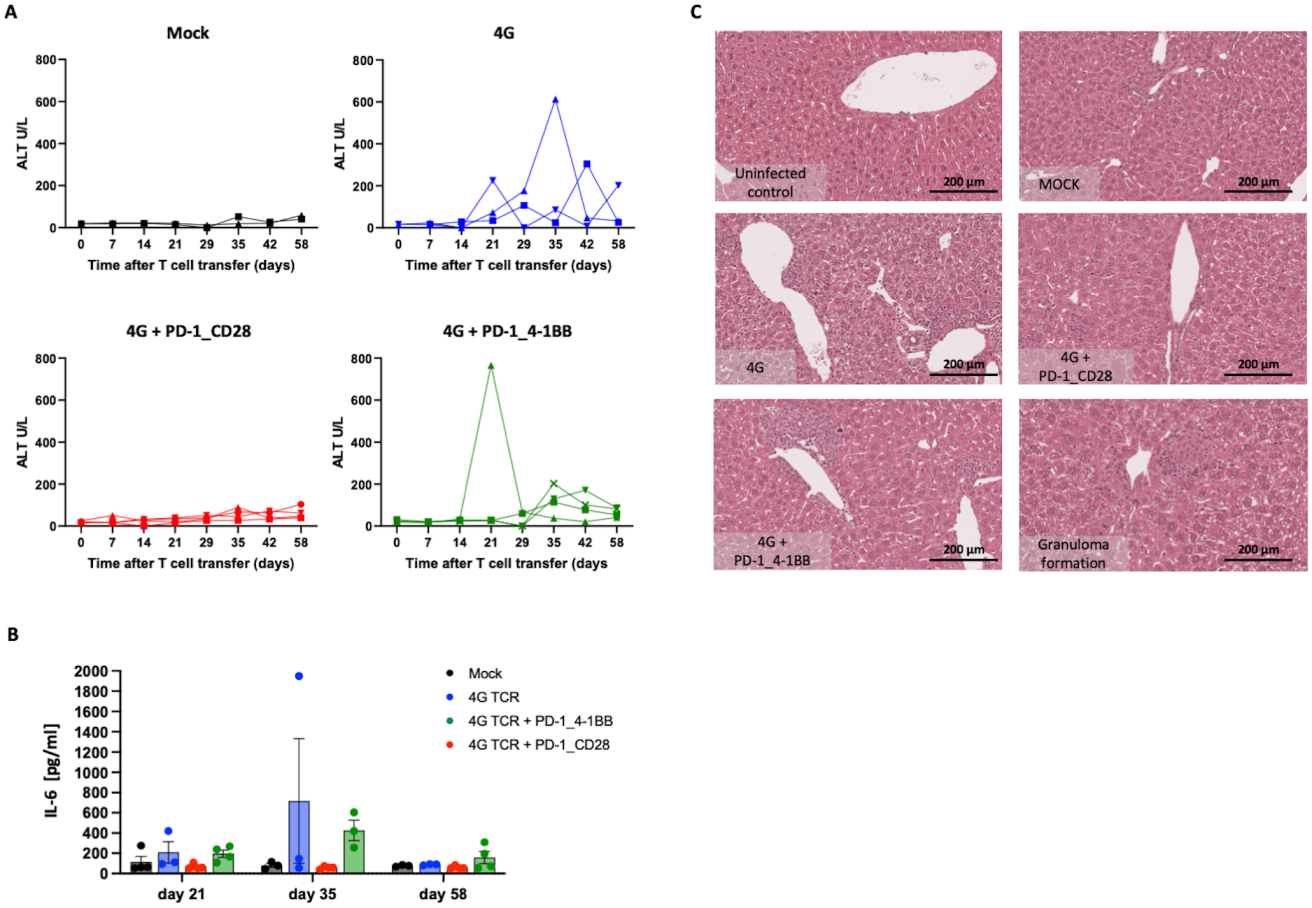
Evaluation of toxicity parameters after adoptive cell therapy in mice. Persistent HBV replication and HLA-A*0201 expression were established in Bl6.Rag1-/- mice using AAV-HBV/AAV-HLA-A2. 0.2x10^6^ CD45.1^+/+^ syngeneic T cells expressing the 4G TCR alone or in co-expression with murine PD-1_CD28 or PD-1_4-1BB immunoswitches were transferred into each mouse at week 6 after AAV-HBV/AAV-HLA-A2 infection (n=5 per group). 4 animals served as mock controls. End-point analyses were performed at day 58. **(A)** Kinetics of ALT levels in serum. **(B)** Plasma IL-6 levels in mice were measured at days 21, 35, and 58 after T-cell transfer. Color bars represent the different treatment setups, dots within each column represent individual mice, and Bars represent mean values of technical triplicates for each sample. **(C)** Representative hematoxylin/eosin stainings of liver slices collected at day 58 after T-cell transfer were evaluated by a pathology expert. Inflammation and T cell infiltration were assessed for all treatment groups and controls. An exemplary granuloma formation is depicted as well. The scale bar represents 200 μm.

Transferred TCR T cells expressing only the TCR, or the TCR in combination with the PD-1_4-1BB immunoswitch, caused transient liver damage, as evidenced by a moderate rise in serum ALT levels in individual mice starting at day 21 (**Figure 7A**). Mock T cells or T cells co-expressing the PD-1_CD28 immunoswitch receptor consistently had low ALT levels.

The mice did not present observable signs of distress or illness throughout the experimental period. IL-6 levels in the blood were analyzed at three time points after T-cell transfer (days 21, 35, and 58), showing mildly elevated levels of 200–600 pg/mL in the groups that cleared the infection (4G and 4G + PD-1_4-1BB) (**Figure 7B**). Furthermore, H&E staining showed that liver tissues from mice treated with HBV-specific T cells exhibited mild to moderate inflammation and T-cell infiltration. A characteristic granuloma formation was observed only in groups treated with TCR-T cells expressing the immunoswitches, indicating increased T cell activity compared to TCR-only T cells (**Figure 7C**). No considerable systemic toxicity or nonspecific reactions were observed.

These findings suggest that T cells expressing the immunoswitches better preserved their effector capacity *in vivo* and were less prone to exhaustion. While both immunoswitch receptors could modulate the exhaustion of TCR T cells, PD-1_4-1BB-expressing TCR-T cells persisted at higher numbers in the liver and showed improved HBV control, as evidenced by reduced HBsAg and HBeAg levels.

### 3.6 PD-1 immunoswitches maintain antigen responsiveness upon multiple rechallenges

**Figure 8:**
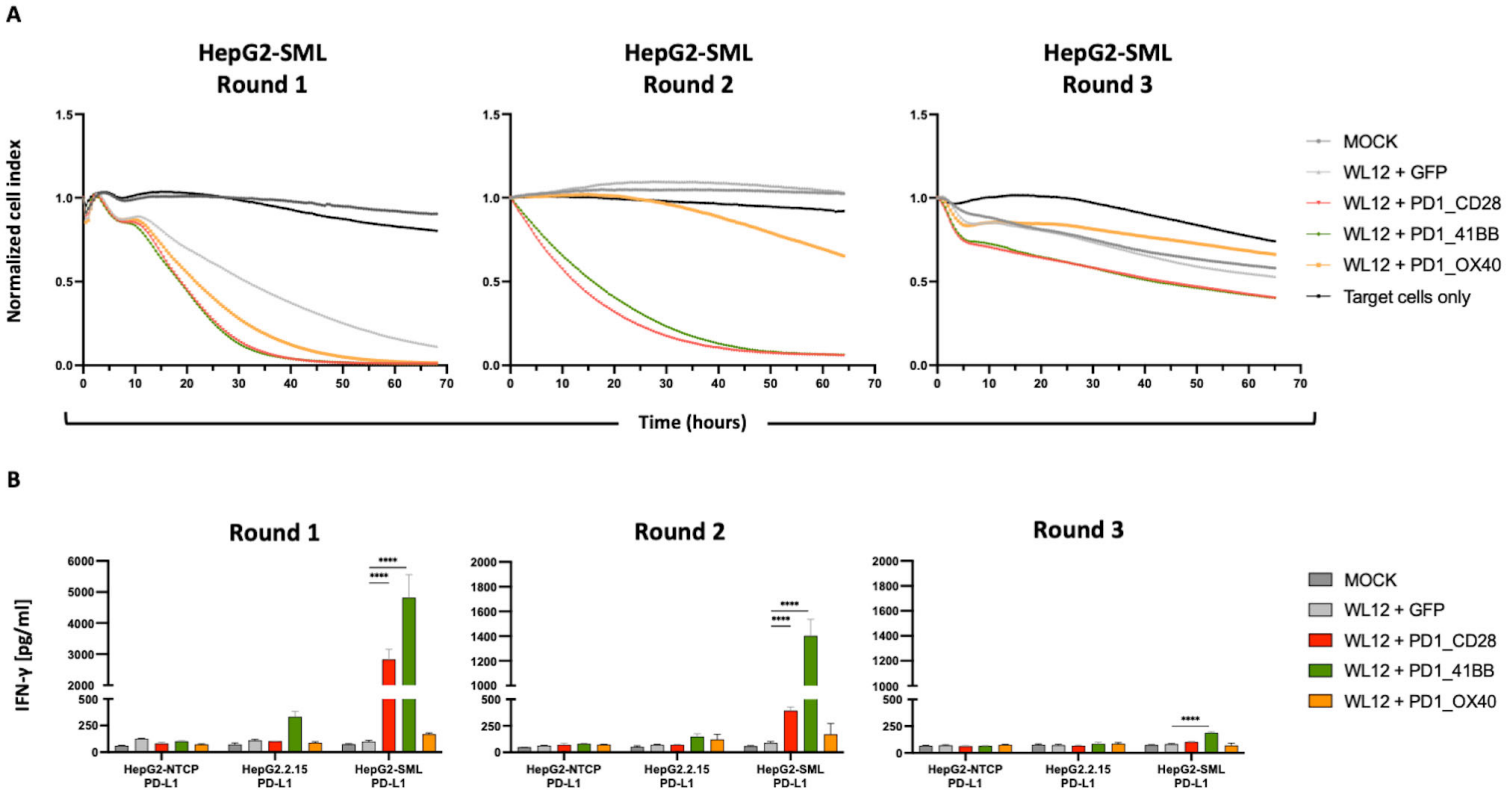
Co-stimulation through PD-1 immunoswitches maintains antigen responsiveness upon multiple rechallenges. HepG2-derived target cell lines (HepG2-NTCP, HepG2.2.15, and HepG2-SML) with or without PD-L1 surface expression were differentiated for 3 days using DMEM differentiation medium and seeded 3 × 10^4^ cells/well in a 96-well electronic microtiter plate (ACEA Biosciences). WL12 TCR T cells co-expressing each individual PD-1-based immunoswitch receptor were added at a 1:1 E:T ratio. T cells expressing only the WL12 TCR (light grey) or no TCR (Mock – dark grey) were used as controls to evaluate the effect of the PD-1_CD28 (red), PD-1_4-1BB (green), or PD-1_OX40 (orange) immunoswitches. Electrical impedance was measured every 30 minutes for 72 hours using the xCELLigence device. After this time (co-culture round), T cells were harvested from each well and transferred to a newly seeded target cell plate for a second co-culture round. The same procedure was performed for a third round. After each co-culture round, killing capacity was analyzed, and supernatants from triplicate wells were collected for downstream analysis. **(A)** Graphs depicting the killing capacity for each co-culture condition are represented using the overtime-normalized cell index data (normalized to the time point of co-culture start) provided by the xCELLigence device. **(B)** IFN-γ secretion was analyzed using the collected supernatants from each co-culture round. Colored bars represent the values for each T cell type condition and follow the same pattern as for the lines on the xCELLigence graphs above. Shown are the mean values of triplicates from one representative experiment repeated 2 times. Statistical analysis was performed using a 2-way analysis of variance (ANOVA) with Dunnett’s multiple-comparison correction. *P-*values represented as follows: <0.0001 (****).

To evaluate T cell response persistence, real-time cytotoxicity assays were performed using differentiated HepG2-SML target cells for a series of antigen rechallenge experiments. While the WL12 TCR-T cells rapidly lost their killing capacity upon antigen rechallenge, cells co-expressing the PD-1 immunoswitch receptors maintained antigen-dependent killing. All three immunoswitch receptors PD-1_CD28, PD-1_4-1BB, and PD-1_OX40) accelerated the killing capacity of WL12 TCR-T cells in the first round of co-culture, indicating stronger early cytolytic capacity. Consistently, TCR-T cells with the PD-1_CD28 and PD-1_4-1BB immunoswitches mediated the fastest target-cell killing, whereas PD-1_OX40 provided intermediate but still superior support relative to TCR-T cells without an immunoswitch receptor (**Figure 8A**).

During the first re-challenge (round 2), T cells carrying only the WL12 TCR completely lost their killing capabilities. In contrast, PD-1_CD28- and PD-1_4-1BB co-expressing TCR-T cells maintained their killing capacity with minimal functional loss. PD-1_OX40-carrying TCR T cells lost most of their killing activity upon the second antigen encounter. In a third round of antigen encounter, the killing capacity of all TCR-T cells was notably lower, even for T cells co-expressing the PD-1 immunoswitches, but for PD-1_CD28 and PD-1_4-1BB, it remained better than that of the controls (**Figure 8A**).

Cytokine analysis further corroborated these findings. IFN-γ levels in supernatants from each co-culture round persisted in immunoswitch-bearing T cells compared to controls expressing only the WL12 TCR. PD-1_4-1BB preserved T-cell functionality best and showed the highest IFN-γ secretion upon antigen re-challenge (**Figure 8B**). The concordance between killing kinetics and cytokine secretion confirmed that PD-1 immunoswitches potentiate both the magnitude and durability of the T-cell response, with PD-1_4-1BB superior to PD-1_CD28 and PD-1_Ox40, which had only a minor effect.

### 3.7 PD-L1 can trigger PD-1_4-1BB immunoswitch signaling in TCR-T cells in trans upon interaction with neighboring cells

**Figure 9:**
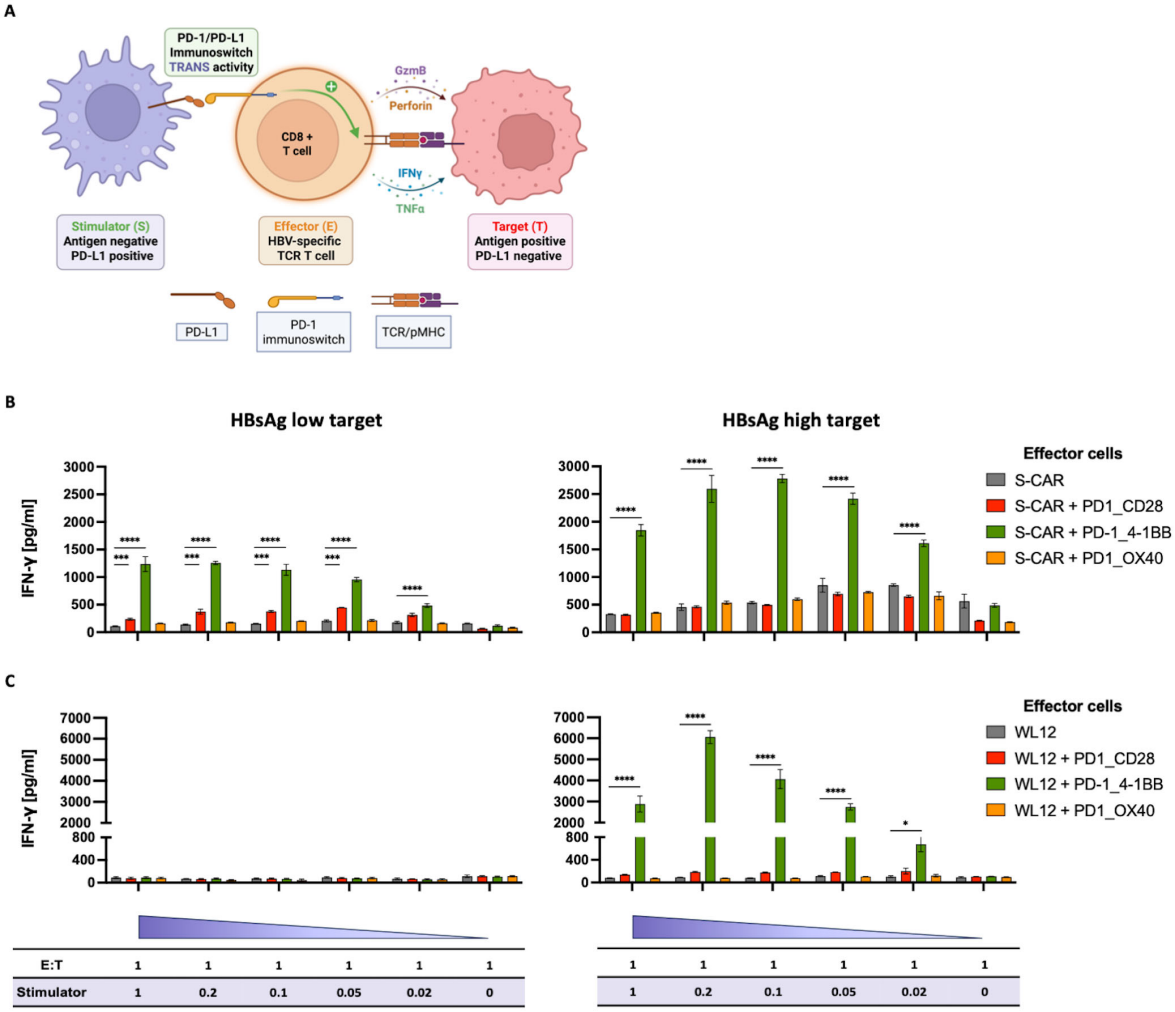
Trans-activity of PD-1_4-1BB immunoswitch can overcome the absence of PD-L1 expression on antigen-expressing hepatocytes **(A)** Schematic drawing of the PD-L1 trans signaling co-culture assay exemplified for TCR+ effector cells. Created in BioRender. Olguin, (L) (2026) https://BioRender.com/xgkvvao. WL12 TCR and S-CAR T cells expressing the PD-1_CD28 (red), PD-1_4-1BB (green), or the PD-1_OX40 (orange) immunoswitches or only the TCR or CAR (grey) were co-cultured with the antigen-expressing PD-L1 negative target cells, HepG2.2.15 and HepG2-SML. Additionally, a third stimulator (S) cell line was included in the co-culture, which is antigen-negative but PD-L1-positive. Stimulation of effector (E) TCR or CAR cells is provided by antigen-positive PD-L1-negative target cells (T), while the PD-L1 ligand for the immunoswitch is delivered through the interaction with antigen-negative, PD-L1-positive neighboring stimulator cells (S). E:T ratio remained at a constant 1:1 ratio (3.5x10^4^ cells), while the amount of stimulator cells (E/T:S ratio) was reduced progressively from 1:1, 1:0.2, 1:0.1, 1:0.05, 1:0.02 until 1:0. IFN-γ was measured after 24 hours in supernatants of the different co-culture conditions for S-CAR **(B)** and TCR **(C)** effector cells using the human IFN-γ ELISA kit. Shown in the graphs are the mean values of triplicates from one representative experiment repeated 3 times. Statistical analysis was performed using a 2-way analysis of variance (ANOVA) with Dunnett’s multiple-comparison correction. *P-*values represented as follows: 0.0332 (*), 0.0002 (***), and <0.0001 (****).

In the liver, PD-L1 is primarily expressed by hepatic sinusoidal endothelial cells and liver macrophages (Kupffer cells), rather than by HBV-infected hepatocytes. To evaluate whether an immunoswitch receptor can be activated in *trans*, a co-culture assay was established to assess the influence of antigen-negative cells, serving as a source of PD-L1, on the stimulation of immunoswitches on HBV-specific T cells (TCR or CAR) before interaction with antigen-positive, PD-L1-negative target cells (**Figure 9A**). Co-cultures of CAR- or TCR-T cells co-expressing the different PD-1-based immunoswitch receptors were set up using PD-L1-negative (low- and high-antigen-expressing) target cells. PD-L1-positive, antigen-negative “stimulator” cells were added at different ratios to evaluate the trans activation threshold of the transduced T cells in the presence or absence of PD-1 immunoswitches.

Both CAR- and TCR-expressing T cells exhibited functional activity (as measured by their capacity to secrete IFN-γ) only when co-expressing the PD-1-based immunoswitches, with the PD-1_4-1BB variant delivering a significantly greater functional improvement. This was observed in CAR T cells regardless of the antigen expression levels (low or high) (**Figure 9B**), while TCR T cells co-expressing the PD-1_4-1BB immunoswitch showed improved IFN-γ secretion only in the context of high antigen-expressing target cells (**Figure 9C**).

These experiments showed that TCR-T cells expressing the PD-1_4-1BB immunoswitch can be activated, even when the PD-L1 ligand is not expressed on the same cell as the target antigen. Hereby, the co-stimulation provided in trans was very efficient, requiring only very few PD-L1-positive cells.

## 4 Discussion

Chronic hepatitis B (CHB) and hepatitis B virus–associated hepatocellular carcinoma (HBV-HCC) remain major causes of liver-related morbidity and mortality despite the availability of effective antiviral therapies, which suppress viral replication but rarely achieve functional cure. A defining immunological hallmark of both disease entities is the persistence of dysfunctional HBV-specific T cells within the liver, an organ intrinsically biased toward immune tolerance and enriched in inhibitory ligands, suppressive myeloid populations, and metabolically restrictive conditions. In HBV-HCC, these constraints are further compounded by tumor-driven immune evasion, including altered antigen presentation, stromal remodeling, and upregulation of immune checkpoints. Therapeutic strategies aiming to restore antiviral and antitumor immunity must therefore not only confer antigen specificity but also sustain T cell functionality under chronic stimulation. Adoptive T cell therapies based on CARs or HBV-specific TCRs represent promising approaches to selectively target infected hepatocytes and HBV-driven malignant cells. However, durable efficacy in CHB and HBV-HCC critically depends on the quality, timing, and topology of co-stimulatory signals delivered to engineered T cells. In the present study, we systematically compared two distinct strategies to enhance HBV-specific T cell function: structural augmentation of a second-generation HBV S-specific CAR (S-CAR 28z) with additional intracellular co-stimulatory domains, and modular, spatially separated co-stimulation delivered via PD-1–based immunoswitch receptors. Our data demonstrate that these approaches yield fundamentally different outcomes, identifying receptor architecture, ligand topology, and signaling pathway selection as decisive parameters shaping engineered T cell performance in the chronically infected liver.

Second-generation CARs incorporating CD28 and CD3ζ signaling domains have shown robust antiviral activity in preclinical HBV models and remain among the most clinically validated CAR designs (14, 28). In principle, the incorporation of additional co-stimulatory domains such as OX40 or 4-1BB could further enhance T cell proliferation, persistence, and effector function, particularly under conditions of prolonged antigen exposure characteristic of CHB. However, our results show that structural incorporation of additional co-stimulatory domains into the intracellular tail of the S-CAR does not improve—and can even impair—antiviral activity. CARs containing OX40 performed comparably to the parental 28z construct, whereas CARs incorporating 4-1BB consistently exhibited reduced cytotoxicity and diminished IFN-γ secretion against HBV-infected HepG2-NTCP target cells. These findings underscore a key principle in CAR biology: functional output is determined not only by signaling domain identity but also by their spatial arrangement, orientation, and distance from the plasma membrane. Third-generation CARs enforce non-physiological clustering of multiple signaling motifs within a single receptor, diverging from the spatial organization of signals within the native immunological synapse. Accumulating evidence indicates that such signal stacking can lead to altered phosphorylation kinetics, tonic signaling, or dysfunctional activation states rather than additive benefit (26, 39, 40). Beyond biological considerations, iterative CAR redesign also carries substantial practical limitations, as each structural modification requires extensive validation, increases developmental complexity, and introduces uncertainty into functional outcomes. These challenges are particularly relevant for HBV infection and HBV-HCC, where antigen density and inhibitory cues vary spatially across infected liver tissue, the tumor core, the invasive margin, and the surrounding non-malignant parenchyma. Together, these observations motivated the exploration of an alternative paradigm that preserves a validated antigen receptor while delivering co-stimulation in a more physiological and adaptable manner.

Physiological T cell activation relies on the integration of spatially distinct signals: antigen recognition through the TCR and co-stimulation delivered by independent receptors localized within the immunological synapse but not fused to the TCR. CARs, in contrast, artificially merge these signals into a single molecule. PD-1–based immunoswitch receptors decouple antigen recognition from co-stimulation while simultaneously exploiting and mitigating a dominant inhibitory pathway in chronic liver disease. These receptors consist of the extracellular domain of PD-1 fused to intracellular signaling domains derived from CD28, 4-1BB, or OX40, and upon engagement with PD-L1, they convert inhibitory signals into productive co-stimulatory cues. Co-expression of PD-1 immunoswitches with the HBV-specific S-CAR 28z significantly enhanced cytotoxicity and IFN-γ secretion, with effects most pronounced at low antigen density, suggesting that immunoswitch-mediated signaling effectively lowers activation thresholds and supports responses in settings that typically favor T-cell dysfunction. Critically, immunoswitch-mediated enhancement remained strictly dependent on PD-L1 expression, confirming that co-stimulation was conditional and spatially restricted rather than constitutive. This modular strategy preserves the validated structure and performance of the parental CAR, enables flexible pairing of a single antigen receptor with different co-stimulatory modules, and (rather than attempting to overcome immune suppression) repurposes PD-L1–rich environments as sources of conditional co-stimulation. In the context of CHB and HBV-HCC, where PD-L1 expression is abundant and persistent on liver sinusoidal endothelial cells, Kupffer cells, and infiltrating immune cells (8, 10, 41, 42), this property is particularly well suited.

The benefits of modular co-stimulation extended beyond CAR-engineered cells to T cells expressing HBV-specific TCRs. TCR-based approaches are especially attractive for HBV-HCC, as HBV-derived peptides can be presented by tumor cells and infected hepatocytes on HLA molecules, even when surface antigen expression is low or heterogeneous (13, 28, 34). However, TCR efficacy is strongly influenced by functional avidity and is profoundly suppressed by PD-1/PD-L1 signaling in the liver (7, 8, 41–43). Both high-avidity (4G) and low-avidity (WL12) HBV-specific TCR T cells exhibited enhanced killing and cytokine secretion when co-expressing PD-1 immunoswitch receptors, with the most pronounced benefit observed in the low-avidity, low-antigen setting. Experiments performed to demonstrate the influence of PD-1/PD-L1 blockade confirmed that the increased T-cell activity corresponds to the interaction of the PD-1-based immunoswitches with PD-L1 expressed on target cells. This suggests that checkpoint blockade in settings where saturation levels of the antibodies are not reached may benefit from combination with PD-1 immunoswitch engineered T cells, through blocking the PD-1/PD-L1 inhibitory pathway while providing additional co-stimulatory signals to enhance T-cell activity.

Mechanistic insight was provided by Jurkat reporter assays, which revealed distinct signaling profiles among immunoswitch variants. PD-1_CD28 preferentially activated NFAT alongside NF-κB and AP-1, consistent with classical CD28-driven effector differentiation, rapid cytokine production, and glycolytic metabolism. In contrast, PD-1_4-1BB and PD-1_OX40 induced stronger NF-κB and AP-1 activation with comparatively reduced NFAT dominance, aligning with known roles of TNFR-family co-stimulation in promoting mitochondrial biogenesis, oxidative phosphorylation, and long-term T cell persistence (26, 44–47). Reduced NFAT dominance is particularly relevant in chronic stimulation contexts, where sustained NFAT signaling without balanced co-stimulation contributes to anergy and exhaustion (7, 41, 43, 48, 49).

The *in vivo* relevance of these findings was evaluated in a chronic HBV infection model. While both TCR-only and immunoswitch-expressing TCR-T cells reduced viral antigens and HBV-DNA levels, PD-1_4-1BB–modified T cells exhibited superior persistence in the liver. Their enhanced recovery was accompanied by reduced expression of exhaustion-associated markers, including the transcription factor TOX, PD-1, and Lag-3. TOX has been identified as a central transcriptional enforcer of T cell exhaustion and epigenetic fixation in chronic infection and cancer (44, 45, 50), and its selective reduction in immunoswitch-modified T cells suggests that PD-1_4-1BB signaling can reshape long-term differentiation trajectories rather than merely transiently enhance effector function. Notably, IFN-γ production upon *ex vivo* restimulation was highest in the TCR-only group, whereas PD-1_4-1BB–modified cells exhibited a more moderate cytokine output. In chronic infection, however, high IFN-γ production alone does not necessarily correlate with durable control and may reflect monofunctional or terminally differentiated states prone to contraction (7, 43). These observations are consistent with reports that 4-1BB signaling supports survival and metabolic stability of antigen-specific CD8 T cells without accelerating terminal differentiation (10, 44), whereas sustained and excessive CD28 signaling under chronic stimulation may promote a strong initial boost of effector activity but predispose cells to activation-induced cell death (49, 51), providing a plausible explanation for the limited *in vivo* efficacy of PD-1_CD28 despite its strong *in vitro* activity. Off-target activation and systemic secondary effects are concerns in adoptive cell therapy. These are linked to the specificity of the CAR and TCR, where antigen cross-reactivity or tonic signaling can occur. These concerns do not apply to the PD-1 switch receptors, as they are not antigen-specific and induce effects only in concert with an antigen-specific signal. If immunoswitch-expressing T cells do not recognize their corresponding antigen via a specific CAR or TCR, the lone interaction between the immunoswitch receptor and its ligand (PD-L1) will not trigger unwanted T-cell activation. This is demonstrated in co-cultures using antigen-negative, PD-L1-positive (non)target cells (HepG2-NTCP_PD-L1), where no unspecific killing or cytokine release (IFN-γ) was detected in the absence of HBV antigen, despite high PD-L1 expression. Moreover, no systemic toxicity was observed in our *in vivo* mouse model. Liver inflammation was mild to moderate, and blood IL-6 levels remained within the expected limits after adoptive T-cell transfer. Similar events are reported after adoptive T cell therapy treatment in clinical trials, and intervention measures are established to mitigate the symptoms.

A central insight from this study is that the spatial source of PD-L1 critically shapes immunoswitch efficacy. In the liver, PD-L1 is predominantly expressed by liver sinusoidal endothelial cells, Kupffer cells, and infiltrating immune populations rather than by infected hepatocytes or tumor cells themselves (10, 12, 52–55). As a result, PD-1 engagement frequently occurs in trans, outside the tight immune synapse formed between T cells and antigen-positive targets. Our trans-activation assays demonstrated that only PD-1_4-1BB immunoswitch–expressing T cells efficiently translated PD-L1 signals delivered by neighboring antigen-negative cells into functional enhancement. This observation provides a mechanistic explanation for the *in vivo* superiority of PD-1_4-1BB over PD-1_CD28 and highlights a fundamental distinction between co-stimulatory pathways: CD28 signaling is optimized for cis interactions within the immune synapse, whereas 4-1BB signaling is permissive to spatially separated ligand engagement.

Repeated antigen rechallenge assays further underscored the relevance of modular co-stimulation for chronic disease. PD-1 immunoswitch–expressing T cells preserved cytotoxicity and cytokine secretion across multiple stimulation cycles, whereas TCR-only controls rapidly lost function. PD-1_4-1BB–expressing antigen-specific T cells again showed the most durable performance, consistent with its association with metabolic maintenance, survival signaling, and memory formation (26, 44, 50, 56). These findings are highly relevant for CHB and HBV-HCC, where persistent antigen exposure is unavoidable and therapeutic T cells must retain function over extended periods (22, 57, 58). They also reflect recent reports that an immune rheostat in the liver suppresses TCR signaling and leads to 4-1BB upregulation (59), and that HBV-specific T cells can be functionally rescued through 4-1BB activation (27).

Collectively, these results support a shift in design philosophy from signal amplification within a single receptor toward spatially distributed, ligand-regulated co-stimulation. Structural augmentation of a second-generation HBV S-CAR with additional co-stimulatory domains failed to enhance, and at times, impaired T cell functionality, underscoring the limits of traditional third-generation CAR configurations and the primacy of receptor architecture. In contrast, modular, spatially separated co-stimulation delivered through PD-1–based immunoswitch receptors effectively enhanced both CAR- and TCR-engineered T cell responses *in vitro* and supported persistence with reduced exhaustion imprinting *in vivo*, while preserving validated antigen receptors and exploiting dominant inhibitory pathways as conditional sources of activation (29–32). Among the designs tested, the PD-1_4-1BB configuration consistently provided the most favorable balance between effector function, durability, and resistance to exhaustion, and appears particularly well suited to the tolerogenic liver environment, where persistent antigen exposure, prolonged contact with liver sinusoidal endothelial cells, and high PD-L1 expression impose strong immune regulatory pressure on engineered T cells. This strategy therefore offers a rational and forward-looking platform to enhance both the magnitude and durability of antiviral and antitumor responses in chronic HBV infection and HBV-associated hepatocellular carcinoma, enabling inhibitory microenvironments to be repurposed as sources of conditional co-stimulation.

## Data Availability

The raw data supporting the conclusions of this article will be made available by the authors, without undue reservation.
